# Supramolecular multivalency effects enhance imine formation in aqueous medium allowing for dynamic modification of enzymatic activity†

**DOI:** 10.1039/d3sc04128j

**Published:** 2023-09-07

**Authors:** Ferran Esteve, Fidan Rahmatova, Jean-Marie Lehn

**Affiliations:** a Laboratoire de Chimie Supramoléculaire, Institut de Science et d'Ingénierie Supramoléculaires (ISIS), Université de Strasbourg 8 allée Gaspard Monge 67000 Strasbourg France estevefranch@unistra.fr lehn@unistra.fr

## Abstract

Imine formation under physiological conditions represents a challenging reaction due to the strong propensity of aldimines to be hydrolyzed. Herein we disclose the remarkable effect of supramolecular multivalency on increasing imine stability. A family of reactive aldehydes was synthesized bearing supramolecularly-active sites within their structure. The imine formation activity for such aldehydes was evaluated and compared with model aldehydes. The reaction of the best-performing species – containing two carboxylate groups-with a set of amines showed a significant decrease in imine yields as the degree of supramolecular multivalency between sidechains decreased. The reversible conjugation of amino acid derivatives and small peptides was also assayed, with excellent selectivities for the imine formation at the Nα position even in substrates containing competing sites. Preliminary results on protein bioconjugation revealed that a model enzyme could be dynamically inhibited upon reaction with the aldehyde, with its native activity being recovered by displacing the imine bonds with a suitable chemical effector (*i.e.*, acylhydrazide).

## Abstract 2

Supramolecular multivalent effects increase the stability of aldimines under physiological conditions. This approach was used for the dynamic inhibition of carbonic anhydrase, recovering its initial activity in the presence of a chemical scavenger.

## Graphical abstract

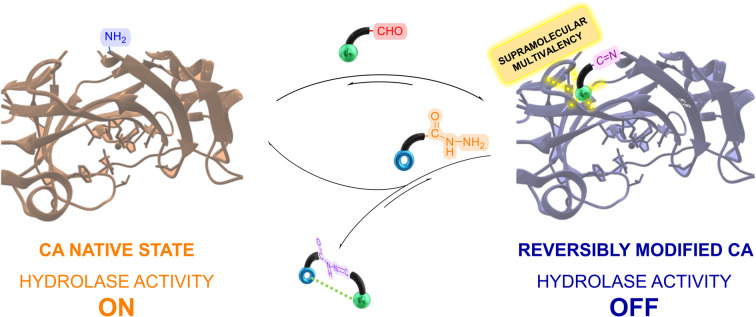

## Introduction

**Fig. 1:**
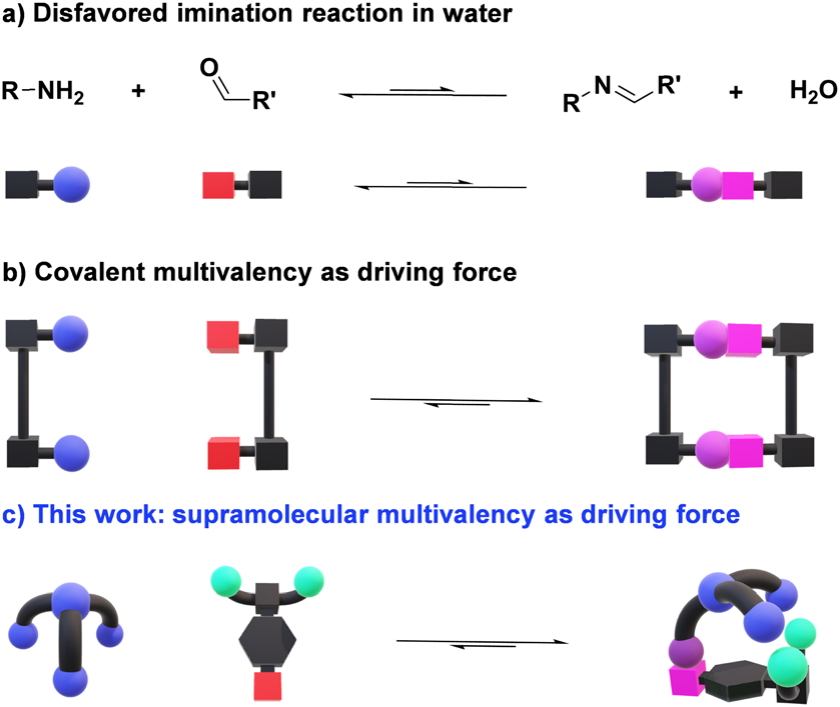
Schematic representations of imine formation in aqueous medium: (a) imination intrinsically disfavored by hydrolysis; (b) increase in imine formation by covalent multivalency; (c) proposed secondary supramolecular interactions as driving force for enhancing condensation reactions in water. Color code: red boxes for aldehyde CHO; blue spheres for amino groups; light green spheres for negatively charged units; purple sphere + pink box for imine bonds.

The design of efficient orthogonal reactions for highly chemo- and regioselective bioconjugations is a major scientific challenge.^1–3^ Dynamic covalent chemistry (DCC), allowing for adaptation through component exchange and selection in response to chemical/physical/environmental agents under equilibrium/out-of-equilibrium conditions,^4,5^ may provide an efficient approach towards this goal.^6–8^ In particular, the widely applicable Schiff base formation reactions represent a promising procedure for the modification of biomolecules.^9^ Whilst the reaction of oximes and hydrazides with naturally occurring aldehydes has been significantly exploited,^10,11^ only a handful of biologically relevant examples relying on aldimine formation have been described.^12–15^ This is a direct consequence of the high tendency of imines to be hydrolyzed in the presence of water, a particularly challenging feature to be overcome (Fig. 1a).^16,17^ Still, the ability of pyridoxal phosphate (PLP) dependent enzymes to form imine bonds *in vivo* infers that a correct design of reactive aldehydes may provide a pathway for performing this reversible chemistry under physiological conditions.^18,19^

Significant progress has been made in the last decade towards efficient aldimine formation in water. For instance, hydrophobic effects have offered many advantages as the lipophilic groups surrounding the imine motifs shield to some extent such reversible bonds from being hydrolyzed.^20–22^ This approach has been further exploited by performing imine condensations in confined apolar arrangements, in which the hydrophobic environments generated within molecular microreactors (*e.g.*, micelles and molecular containers) led to remarkable results.^23–25^ The synthesis of complex 3D-architectures has also been achieved by exploiting multivalency.^26–28^ In these cases, the simultaneous cleavage of multiple dynamic covalent bonds – clearly more challenging than breaking a single bond – further decreases the tendency of imines to hydrolyze in aqueous medium (Fig. 1b).^29^ Despite the promising advances reached, most of the reported systems are only stable at basic pH or in the presence of certain thermodynamic templates, hampering the use of such designs for dynamic biological applications.^30,31^ The most common approach for overcoming these limitations relies on the coupling of fast and site-selective reversible imination steps with irreversible post-modification reactions (*e.g.*, imine reduction), at expense of the dynamic nature of the systems.^32–36^

Considering these precedents, we surmised that the presence of secondary supramolecular forces between the aldehyde and amine sidechains could result in high-yielding condensation reactions (Fig. 1c), even in competitive media. This is indeed a driving force in PLP-mediated biological transformations, with the related aldimine intermediates being stabilized through networks of supramolecular interactions with different units elegantly preorganized in active pockets of enzymes.^37^ Related cooperative non-covalent forces between the two components of the dynamic adduct are here described as supramolecular multivalent effects.

## Rationale

Some key factors must be considered when discussing imination reactions in water. Firstly, imines will only be effectively generated in aqueous solutions when the optimal balance between aldehyde reactivity and solubility is met. Further, aldimine products should not precipitate in the medium, as this would act as a kinetic trap, precluding a satisfactory evaluation of the thermodynamic features of the systems.^38^ One may however remark, that in certain instances, precipitation of the condensate may select for the desired derivative. In addition, hydration of the aldehyde partner competes with imine formation, albeit the same structural factors favoring hydration could also be advantageous for the generation of the imine. It must be noted, however, that whereas the nucleophilic addition of a primary amine would be expected to correlate well with hydration, the subsequent water elimination step may show an opposite trend.^17^ Bearing these considerations in mind, we designed a novel family of aldehydes, namely A7, A8, and A9 (Scheme S1†) derived from the quite reactive precursor 2,3,4,5,6-pentafluorobenzaldehyde (A6) to study the role of supramolecular interactions in accomplishing high imine yields under physiological conditions.^39^ One may predict that such supramolecular multivalency might be strong enough to enhance the thermodynamic stability of imines over hydrolysis (Fig. 1c).^40,41^

## Results and discussion

### Evaluation of imine formation activity and of attractive supramolecular forces

**Fig. 2:**
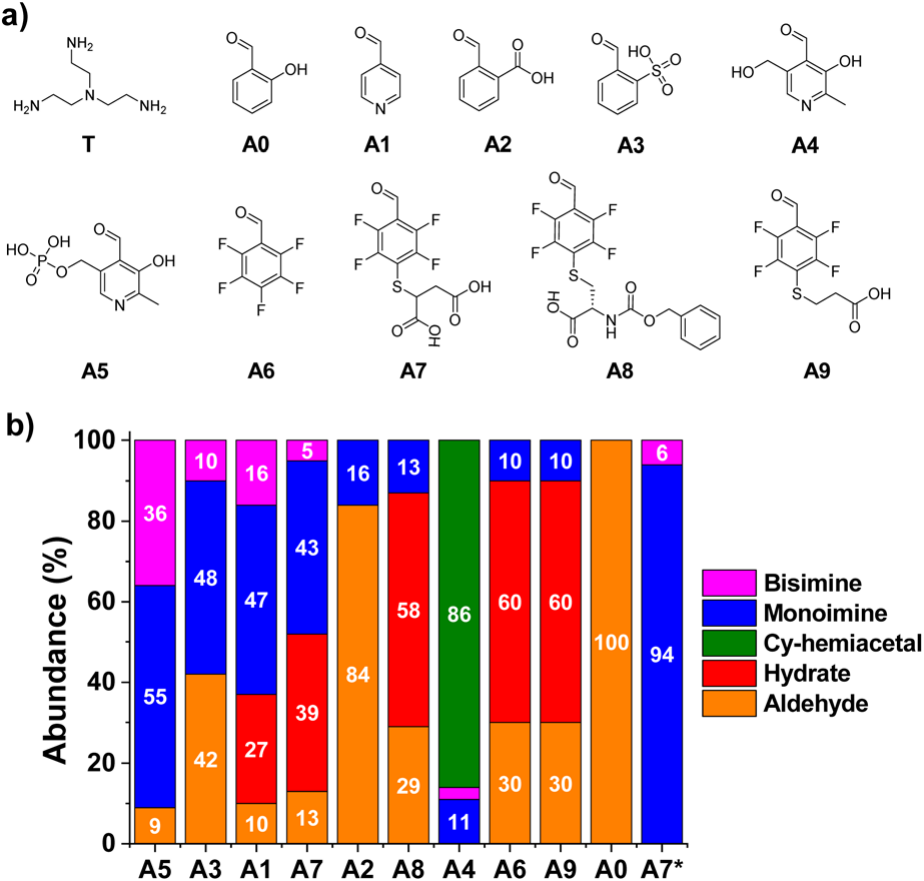
(a) Chemical structures and (b) component abundance (%) as calculated from ^1^H NMR spectra (500 MHz, 295 K, D_2_O) for the tested aldehydes upon reaction with T. Reaction conditions: D_2_O (pD = 7.2, phosphate buffer 50 mM), 295 K, 1 h, [AX] = [T] = 5 mM. A7*: [A7] = 5 mM, [T] = 50 mM. Cy-hemiacetal: cyclic hemiacetal after intramolecular ring-closing condensation of A4. Aldehydes sorted with decreasing values of monoamine abundances. The abundances of the bisimines were calculated considering the integration of two equivalent CHO protons.

To evaluate the relative efficiency of imine formation, the aldehydes A0–A5 were selected and their activity was investigated in comparison to the results obtained for A6–9. A few earlier studies have shown that aldehydes derived from pyridine (A1, Fig. 2) or aldehydes that contain strongly electron-withdrawing units in *ortho*-position to the carbonyl group (A3, Fig. 2) can lead to high activities towards the formation of imines in aqueous media.^14,39,42,43^ Two pyridoxal-derided species, namely A4 and A5, were also considered as such compounds are known to yield highly efficient Schiff base formation. The tris-amine TREN (T) was selected as model amine to test multivalency since the presence of additional positively charged moieties in the structure, +2 or +3 depending on the pH,^44^ serves as an optimal scenario for assaying the role of secondary electrostatic interactions between the pendant arms of the two components of the imine (*i.e.*, aldehyde and amine). Besides, its chemical properties can showcase the reactivity differences between the terminal-amino group and Nε-sites of Lys residues present in proteins.^45,46^

Condensation reactions were followed by the appearance of the imine CH signal in the ^1^H-NMR spectra shifted to lower *δ* with respect to the peak of the parent aldehyde. The relative abundance of imine and hydrate were determined by the integration of aldehyde and imine/hydrate peaks.^17^ The two reagents were mixed in a 1 : 1 molar ratio using a phosphate buffer (50 mM) for maintaining a pH value similar to physiological conditions (pD = 7.2). The equilibrium state was reached in most of the cases within the first 10 min of reaction, in accordance with the fast rates of imine formation previously reported.^47^ Notwithstanding, all reaction crudes were also monitored after 1, 24 and 48 h to assure that equilibrium was attained by noting only negligible changes in integration. Product abundances in Fig. 2 show interesting results in terms of structure–reactivity relationships. Salicylaldehyde (A0) did not react with T under the conditions assayed (Fig. 2). Aldehydes A1 and A3 resulted in conversions of *ca.* 60% into aldimine species, although the selectivity for the mono-over the bisimine product was quite low (75 and 83%, respectively, Fig. S1†). Compound A2 showed a 16% yield for the monoimine derivative, but with excellent selectivity (Fig. 2 and S1†). Regarding pyridoxal derivatives, the aldehydes were nearly completely consumed after 1 h of reaction, indicating a clear electrophilic character for their carbonyl groups. Nevertheless, whereas A4 tended to preferentially form the cyclic hemiacetal, PLP (A5) led to >90% imine abundance. The enhanced activity of A5 in comparison to A1–3, however, gave very low selectivity of monoimine species, as shown by the high abundance of the bisimine (A5)_2_T (36%, Fig. 2). Regarding the aldehydes derived from A6, the nature of the sidechain was clearly affecting the imination outcome. Aldehyde A6 was preferentially forming the hydrate derivative (60%), with only 9% of imine A6T being detected. When only one carboxylate anion was present in the structure (A8 and A9), the aldimine yields were of *ca.* 10%, with excellent selectivities towards the monoimine derivatives. Satisfactorily, the reaction of T with A7 (containing two anionic groups) led to good imine yields (48%, Fig. 2) with high selectivity for the mono-functionalized Schiff base (91%, Fig. S1†). As expected, increasing the equivalents of the triamino compound shifted the equilibrium towards the formation of the A7T monoimine product, with remarkable yield and selectivity (both values >90%, A7* in Fig. 2 and S1†). These preliminary results suggested that the imine stability was increased significantly by complementary electrostatic attractive forces between the carboxylate and protonated amine groups of the multivalent sidechains, as well as by a contribution of the local concentration of reactive groups.

**Fig. 3:**
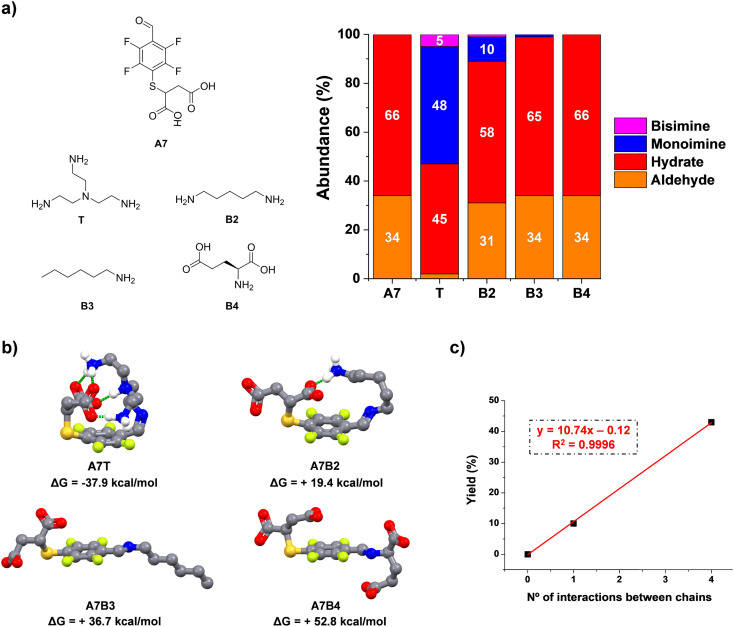
(a) Chemical structures and component abundances (%) for reactions between aldehyde A7 and model amines T/B2–4. Abundances calculated from ^1^H NMR spectra (500 MHz, 295 K, D_2_O). Reaction conditions: D_2_O (pD = 7.2, phosphate buffer 50 mM), 295 K, 1 h, [A7] = [amine] = 5 mM. The abundances of the bisimines were calculated considering the integration of two equivalent CHO protons. (b) DFT (b3lyp/6-31g(d,p); solvent = water) optimized geometries for the aldimine compounds. Free-energies of formation (kcal mol^−1^) have been calculated using the free-energies of precursors and the water molecule released in each condensation. Hydrogen bonds/electrostatic forces between the two pendant arms of the building blocks have been highlighted with discontinuous green lines. Non-essential hydrogens have been omitted for clarity. (c) Linear fitting correlating the number of interactions between the sidechains and experimental yields attained in the imination reaction.

In the light of these results, we studied the condensation reaction between A7 and model amino compounds containing different number of charges (T and B2–4, Fig. 3a). Thus, the reaction between A7 and B2 yielded a noticeable decrease in aldimine formation (43 *vs.* 10% for A7T and A7B2, respectively). In a similar manner, hexylamine (B3) gave only traces of imine under the same conditions (<1%, Fig. 3a). In this case there was no additional electrostatic interaction for promoting imine formation. As surmised, when A7 was reacted with B4 (each of them containing two negative charges on the residues) no imino-compound was detected even after 48 h of equilibration, likely due to the strong repulsion between the four negatively charged units. These results are in line with an increased stability of the imine species through supramolecular multivalency.

To corroborate the effect of secondary supramolecular interactions, DFT calculations were carried out for the different aldimine compounds, *viz.*A7T, A7B2, A7B3, and A7B4. As expected, all the optimized geometries adopted a -*trans* isomeric form for the imine bond. Regarding the non-covalent forces between chains, a significant energy difference was found for the extreme cases A7T and A7B3/A7B4. Whereas the most stable imine A7T adopts a tangled conformation assisted by four intramolecular polar hydrogen bonds, A7B3 and A7B4 rather adopted an expanded spatial distribution imposing large distances between the negatively charged chain of A7 and the hydrophobic/anionic arm of B3/B4 (Fig. 3b). An intermediate scenario was found for A7B2, forming only one intramolecular non-covalent interaction between units. In this case, the maximum yield of aldimine was of 10%, in agreement with the lower thermodynamic stability of this species in comparison with that presenting a higher degree of supramolecular multivalency (A7T). The plot for mono-imine yields *vs.* the number of non-covalent forces between chains revealed a linear trend (Fig. 3c). Therefore, these structural features appear desirable for the reversible bioconjugation of proteins containing a large number of positively charged residues (*e.g.*, Lys/Arg) that provide numerous supramolecular complementary forces with A7.

### Reversible tethering of amino acids and small peptides: site-selective Nα-functionalization

**Fig. 4:**
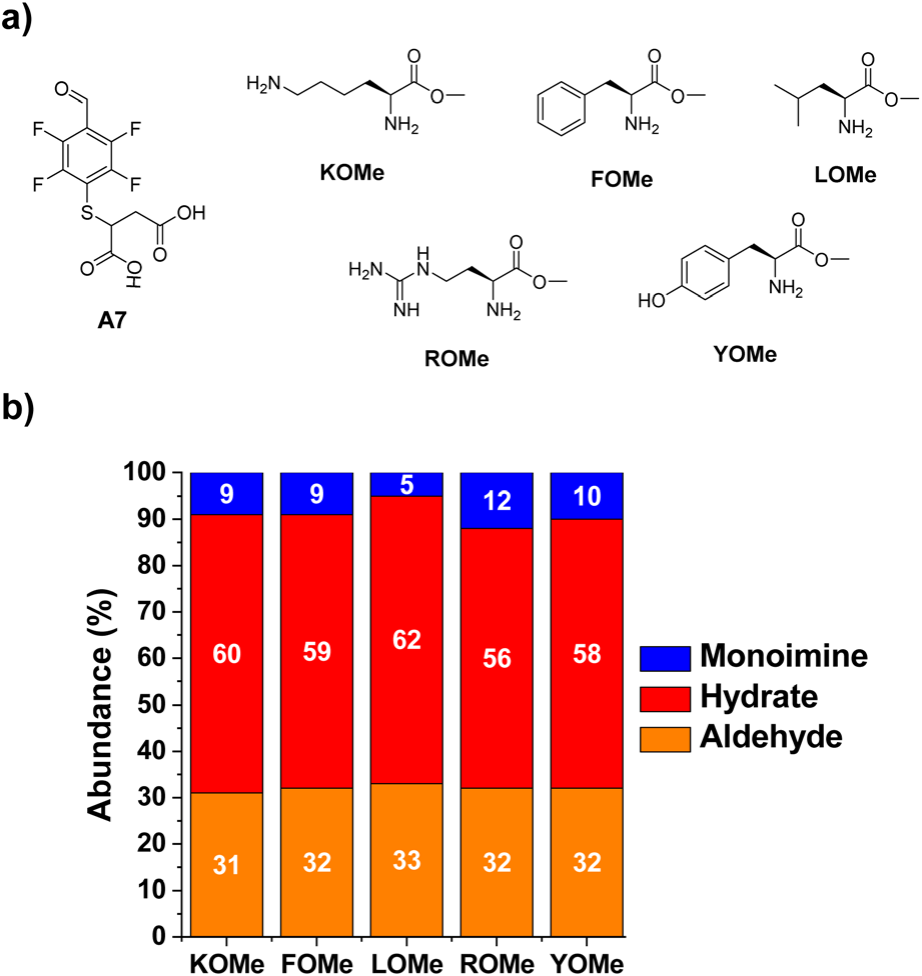
(a) Chemical structures of the aldehyde A7 and the different methyl ester derivatives of selected amino acids. (b) Component abundances (%) as calculated from ^1^H NMR spectra (500 MHz, 295 K, D_2_O). Reaction conditions: D_2_O (pD = 7.2, phosphate buffer 50 mM), 295 K, 1 h, [A7] = [XOMe] = 5 mM.

The next step towards the functionalization of proteins was to evaluate the reactivity of A7 with different amino acid derivatives. The effect of the amino acid residue was studied by screening a set of five different canonical amino acids (Fig. 4a). The methyl ester derivatives (XOMe) were used in all cases to avoid repulsive electrostatic interactions between the corresponding carboxylate anions and those of A7. This aldehyde was able to form the desired imino species in 5–12% yields with the amino acid derivatives considered (Fig. 4b). The highest yield was attained for ROMe (12%), likely as a result of the secondary electrostatic interactions between the charged guanidinium unit of ROME and the carboxylate groups of A7.

**Fig. 5:**
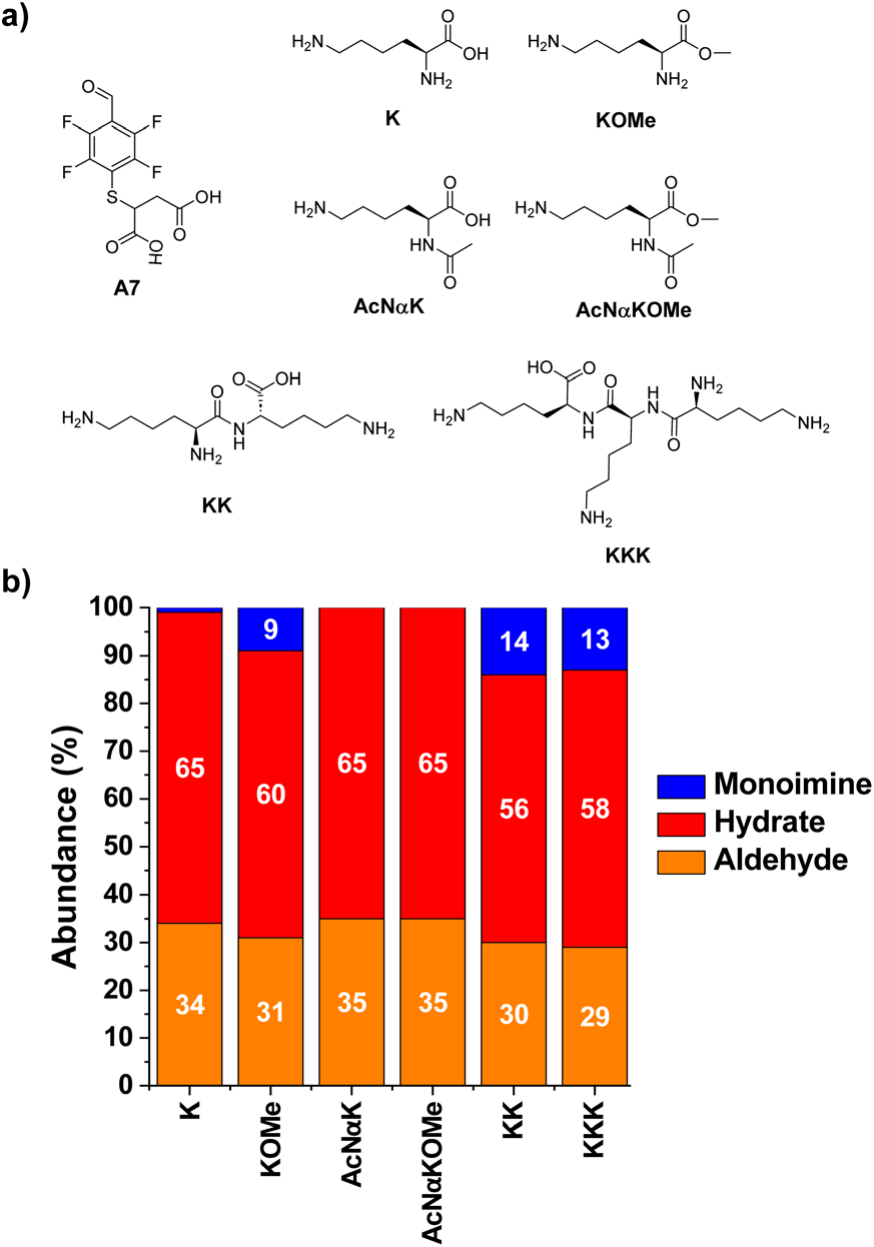
(a) Chemical structures and (b) component abundances (%) as calculated from ^1^H NMR (500 MHz, 295 K, D_2_O) for the tested Lys derivatives upon reaction with A7. Reaction conditions: D_2_O (pD = 7.2, phosphate buffer 50 mM), 295 K, 1 h, [A7] = [XKY] = 5 mM.

The reactions of A7 with several Lys derivatives and small peptides were next examined as diverse recognition sites on the sidechains could provide additional driving forces (Fig. 5). The results agreed well with the proposed role of attractive and/or repulsive secondary electrostatic forces. The reaction between A7 and K did not result in the formation of the corresponding A7K. Removing the negative charge of the carboxylate group in K by using its methyl ester derivative (KOMe) resulted in *ca.* 10% yield of A7KOMe, indicating that the carboxylate group in K was disfavoring the formation of the imine. Only one CH imine signal was detected in the ^1^H NMR spectrum for the reaction between A7 and KOMe, suggesting that the aldimine formation was regioselective. No imine was formed in the reaction between A7 and AcNαK/AcNαKOMe. Hence, the reaction was clearly occurring at the Nα site since when this position was blocked as an acetamide group, no aldimine was observed. This preferential reactivity of the Nα is related to its lower basicity (p*K*_a_ ≈ 6–8) compared to that of lysine ε-amino groups (p*K*_a_ ≈ 10), as previously described.^46^ Its lower basicity leads to a lower protonation of the terminal –NH_2_, enhancing its propensity to act as a nucleophile and form the imine.

The bi- and tripeptidic Lys derivatives, namely KK and KKK, led only to a slight increase in the yields, 14 and 13% respectively. The selectivity for the Nα-aldimine was excellent in all cases, with no additional imino compound detected in solution. As further validation of the reaction site specificity, 2D NMR analyses (HMBC and edited HSQC, Fig. S2†) revealed that the CH of the imine at 8.52 ppm was in close proximity to the chiral carbon of A7KK appearing at 73.7 ppm (Fig. S2b and c†). The most stable conformation calculated by DFT for A7KK inferred the presence of only one attractive hydrogen bond/electrostatic interaction, involving one of the Nε of KK and one of the carboxylate anions of the A7 chain (Fig. S3†). Therefore, the imine yield in the condensation between A7 and KK agrees well with that calculated using the linear correlation proposed in Fig. 3c (experimental: 14%; calculated: 11%). An increase in molar ratio KK/A7 up to 10 eq. of KK gave imine yields >40% and Nα selectivities >99% (Fig. S4†). This remarkable selectivity may be of interest for the selective modification of the –NH_2_ terminal of peptides and proteins.

As chirality may be expected to play a pivotal role in molecular recognition processes,^48,49^ the chiral aldehyde A8 was studied instead of the achiral A7. In all cases, the imine formation was lower than with A7, with the largest effect being observed for ROMe, in agreement with imine stabilization by attractive electrostatic interactions. In contrast, the affinity of A8 to form imines was significantly enhanced when using KK. The molar ratio effect on the final distribution of compounds was also studied once equilibrium was reached after 1 h of reaction. Comparing results obtained with those for A7, the cysteine-derived A8 gave higher imine yields (see for instance Fig. S4 and S6†), while maintaining excellent selectivities for the Nα monoimine A8KK (>99%). When 10 eq. of KK were mixed with A8, A8KK was generated in an NMR yield of 62%. These results paved the way for exploring protein modifications using A7 and A8.

### Bioorthogonal modification of carbonic anhydrase (CA) and dynamic regulation of its activity

Although a great number of important drugs exploit irreversible binding for inhibition, it has been considered that potential long-term toxicity has led to near exclusion from the discovery program.^13,50^ Moreover, the still critical challenge of site-selective protein bioconjugations emphasizes the need to develop novel strategies that can render bioorthogonal and regioselective conjugations.^35,51^ The use of reversible covalent ligation has offered advantages for dynamic protein tethering as dynamic bonds allow for the occurrence of error checking.^52,53^ Promising results in Nα-selective modifications have recently taken advantage of its lower degree of protonation at physiological conditions.^54–56^ Aldimine formation has been proven useful for distinguishing between Nε-sites of Lys residues, although a coupled irreversible nucleophilic attack was required for increasing the stability of conjugated units.^33,34^ In such examples, the microenvironment of each Lys residue was controlling their reactivity and, therefore, the regioselectivity outcome. This is of prior importance when discussing the so-called “hyper-reactive lysines” that present a markedly enhanced Nε-nucleophilicity, presenting p*K*_a_ values ≈ 5 units lower than solvent-exposed analogues.^57^

**Fig. 6:**
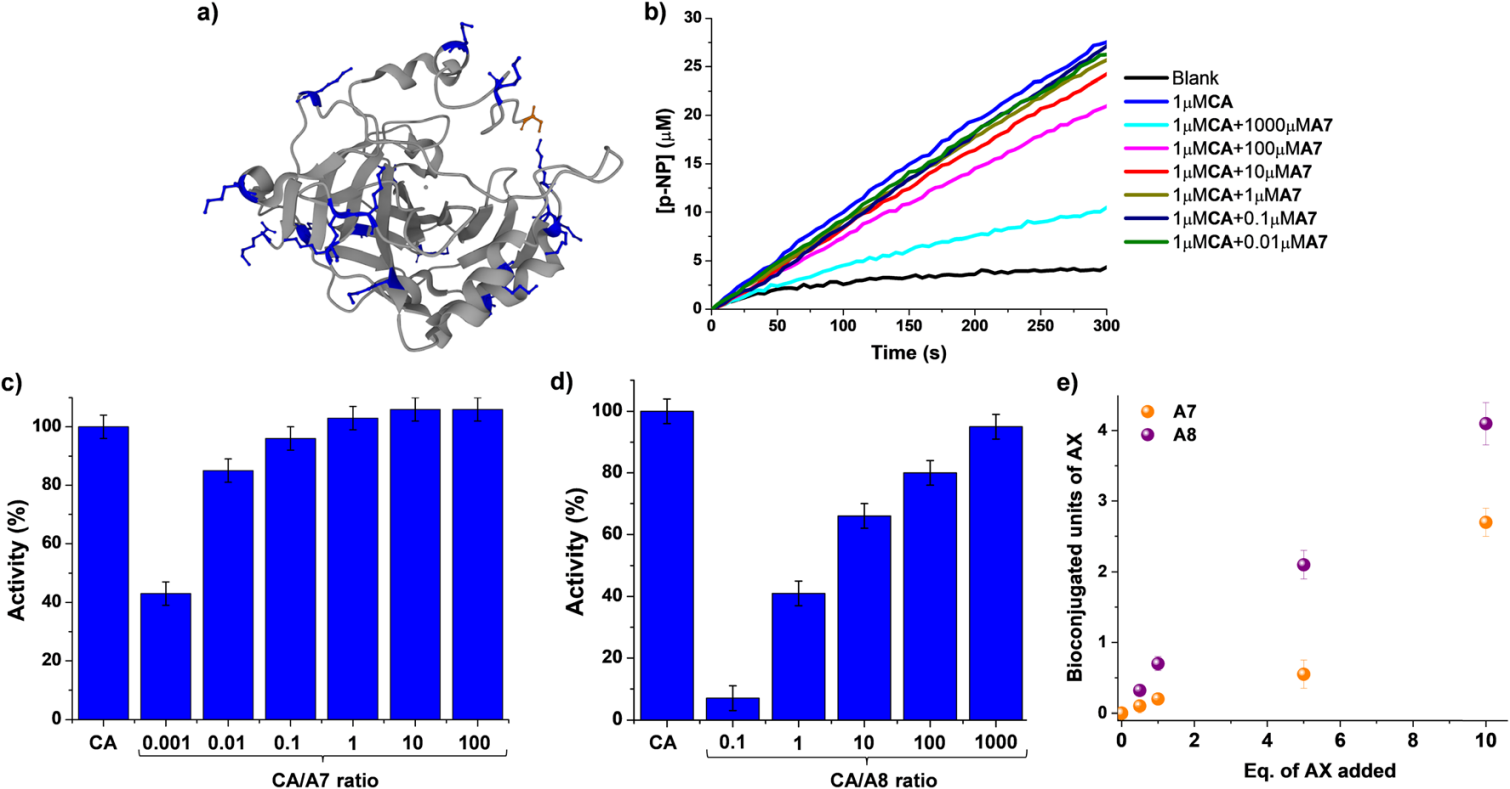
(a) 3D-structure of native CA from bovine erythrocytes (pdb: 1v9e). The backbone of the protein (including α-helices and β-sheets) is colored grey. Lys (18 in total) and terminal Nα sites (S1) are highlighted in blue and orange respectively, using the ball and stick model. (b) UV monitoring (linear range, 0–300 min) of the hydrolysis of *p*-NPA in the presence of CA and different equivalents of A7. Reaction conditions: D_2_O (pD = 7.2), 335 K. Relative activities (%) observed for the different concentrations of A7 (c) and A8 (d) assayed. The activity of the native state CA (1 μM) has been used as reference. (e) Comparison of the MALDI analyses studying the CA bioconjugation efficiency in the presence of A7 and A8. The amount of units incorporated have been determined considering the increase in molecular weight of the CA, the equivalents of each aldehyde added, and the molecular weight of each aldehyde. See Fig. S8† for fitted curves, and S30–40† for full *m*/*z* scale MALDI analyses. MALDI conditions: PNA matrix, D_2_O, pD = 7.2, phosphate buffer. CA concentration = 0.1 mM. Error bars calculated considering the width of the Gaussian distribution for each sample.

As a consequence, we decided to explore whether the activity of a model enzyme, *i.e.* carbonic anhydrase (CA), could be dynamically modified by reaction with aldehydes A7/A8. CA is a ubiquitously found metalloenzyme playing an essential role in the preservation of acid–base homeostasis, pH regulation, and fluid balance, through the interconversion between CO_2_ and bicarbonate ion.^58^ In addition, its isomeric II form (hCAII) is found in many human tissues and is for instance responsible for maintaining inner eye pressure.^59^ Therefore, many efforts have been devoted in the last years to designing adequate inhibitors for CA, as dysregulation of this enzyme is directly related to severe diseases like glaucoma.^60,61^CA also presents esterase activity that facilitates the monitoring of its catalytic performance when interacting with different chemical species.^62^CA from bovine erythrocytes was chosen for evaluating not only the efficiency of the bioconjugations but also their effect on its activity in the formation of *para*-nitrophenol from 4-nitrophenylacetate, *p*-NP and *p*-NPA respectively. One may note that the selection of CA as the model substrate took also into account the presence of 18 solvent-accessible Lys sites (marked in blue in Fig. 6a) that might facilitate the chemical modification with the proposed aldehydes.

UV monitoring of kinetic profiles for the hydrolysis of *p*-NPA catalyzed by 0.1 mol% of CA in the absence and presence of different amounts of A7 revealed a minor effect on its activity (Fig. 6b and c). For instance, introducing 100 equivalents of A7 (*i.e.*, 0.01 CA/A7 ratio) only led to a decrease in activity of *ca.* 15%. The addition of 1000 eq. of A7 was needed for reducing the catalytic activity of CA to ≈50% of its initial value, suggesting that the bioconjugation of A7 was not critically altering the catalytic performance of the enzyme. In contrast, the treatment of CA with compound A8 promoted a strong inhibition of the biomolecule (Fig. 6d and S7†). The apparent half maximal inhibitory concentration (IC50) value was of *ca.* 1 μM, corresponding to a 1 : 1 molar ratio with CA. In the presence of 10 equivalents of A8, the activity of the enzyme was reduced to 7% of its initial value, stressing the inhibiting potential of A8.

MALDI analyses showed an increase in the molecular weight of the biomolecule dependent on the equivalents of AX introduced (Fig. S8†). These results indicate a much stronger effect of A8 than of A7 (Fig. 6e). When 1 eq. of A8 was added to the protein, an increase in molecular weight of *ca.* 300 g mol^−1^ was noted, in accordance with the tethering of 0.7 units of A8.^63^ On the other hand, the introduction of 1 eq. of A7 led to the incorporation of only 0.2 units of A7. Similarly, the use of 5 eq. of the aldehydes resulted in the binding of 2 and 0.5 units for A8 and A7, respectively. At higher excesses of aldehydes (*i.e.*, 10 eq.) 3 and 4 units of A7 and A8 were respectively bound to CA. Neither the CA molecular weight nor its enzymatic activity were altered in the presence of 10 eq. of A0 (Fig. S9†). Therefore, the supramolecular multivalency effects are crucial for the CA dynamic tethering and regulation of its activity. These outcomes point to promising applications of DCC in site-selective bioconjugations since, despite the high frequency of Lys sites in CA, only the thermodynamically favored sites were effectively functionalized even when adding an excess of the reactive species.

The tethering of A8 to CA was studied in greater detail to shed light on the nature of its inhibitory activity. ^1^H NMR titration experiments suggested the appearance of a low-intensity peak at 8.43 ppm, assigned to the formation of aldimine derivatives with the protein (Fig. S10a†). The presence of the CHO signal (9.97 ppm) even at <5 eq. of A8 supports the incomplete bioconjugation (only thermodynamically favored sites), observed in the MALDI analyses. Interestingly, some regions of the protein seemed to experience a noticeable change in the electronic environment as a result of the dynamic modification (Fig. S10b†). Circular dichroism (CD) titration experiments showed a similar scenario, with CA undergoing a conformational modification in the presence of A8 (Fig. S11 and S12†). The negative band centered at 219 nm, ascribed to β-sheet-like arrangements in the native state of the protein, seemed to decrease in intensity with increasing amounts of A8, suggesting that some sections of the β-sheets were being perturbed. Considering the structural features of A8, one might surmise that the inhibition mode of action can rely on allosteric processes, in which the conformational changes induced by aldimine modifications reduced the efficiency of the catalyst. The 3D structure of native CA shows a highly hindered active site located in the center of a conical cavity defined by curved β-sheets (see Fig. S13a†).^64^ The most plausible trajectory for *p*-NPA to reach the active site is its diffusion through the conical cavity, in the entrance of which are located a terminal Ser (S1) group and a Lys residue (K168) (see spacefilling residues in Fig. S13a†). Preliminary docking experiments were carried out for studying the preferential recognition sites for A8 in the CA structure, using SwissDock.^65^ A blind and accurate docking was selected for screening all possible recognition sites. The top-ranked position (binding free-energy: −8.15 kcal mol^−1^) located A8 near the terminal Nα unit and K168, stabilized through several non-covalent forces with peptidic bonds and amino acid residues (Fig. S13b and c†). For instance, the carboxylate group of A8 is tightly bound to S1 through two hydrogen bonds, one with its terminal amino group and one with the NH of the S1–H2 peptidic bond, with distances between heavy atoms of 2.753 and 2.517 Å, respectively. The negatively charged unit of E234 seems to be interacting by means of anion⋯π forces (O_E234_⋯C_ar,A8_ = 3.059 Å) with the electron-deficient tetrafluoro-substituted aromatic ring of A8. Additional CH⋯π interactions between the Cbz-aromatic motif of A8 and the imidazole ring of H3 could be further stabilizing the host–guest adduct (CH_H3_⋯C_ar,A8_ = 2.674 Å). The formation of the aldimine derivative is likely to occur in that specific pocket as a result of the increased local concentration of the nucleophilic scaffolds (Nε-Lys168/Nα-Ser1) and the electrophilic CHO group of A8. Hence, the imine formation between CA and A8 might hamper the diffusion of *p*-NPA to the active site, leading to allosteric regulation of the enzymatic activity.^66^

**Fig. 7:**
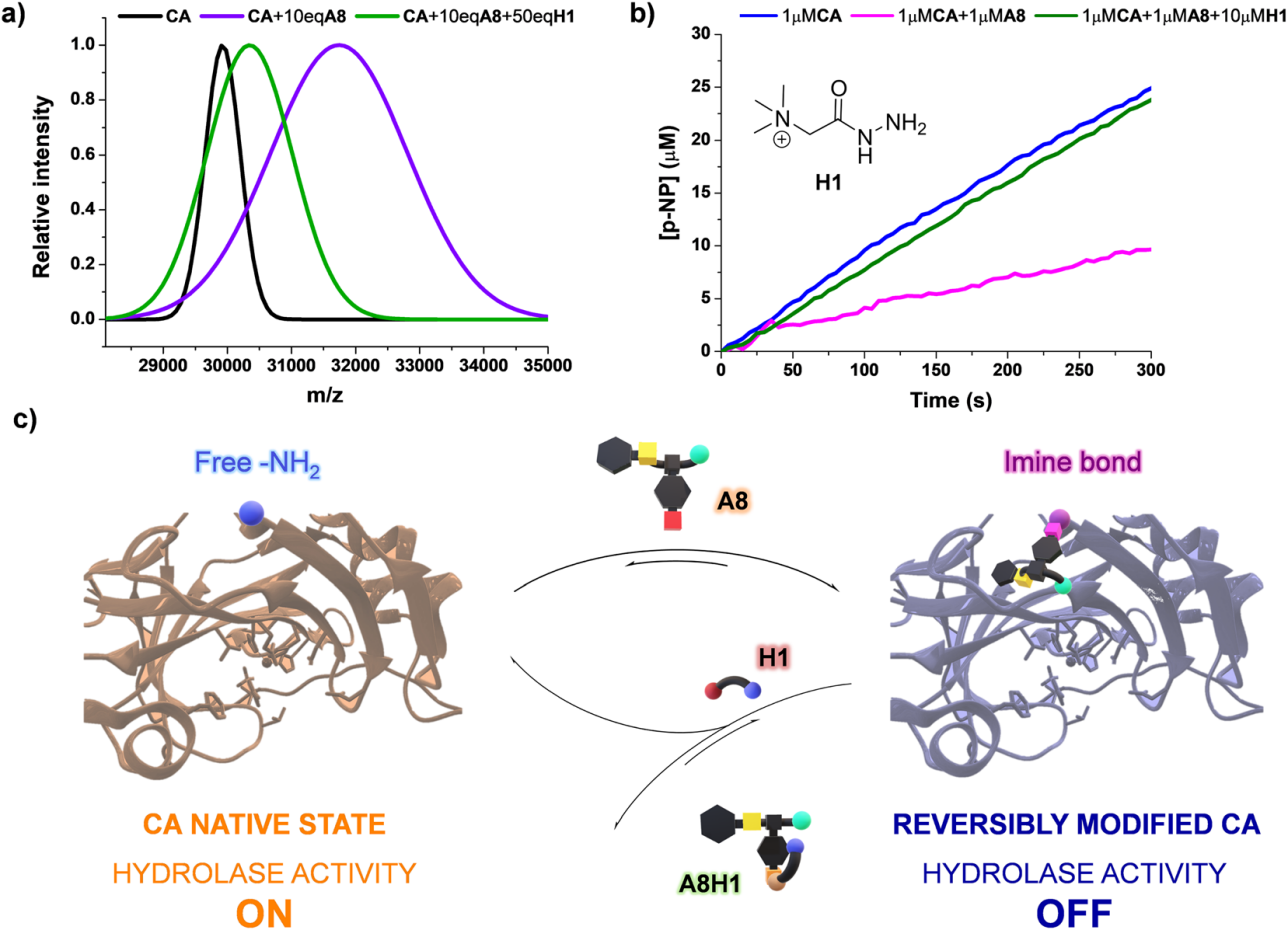
(a) MALDI analyses (PNA matrix, D_2_O, pD 7.2 phosphate buffer) for the bioconjugated (A8)*_x_*CA (0.1 mM) in the presence and absence of effector H1. The peaks were fitted to a Gaussian distribution. The dispersity of the bioconjugation was estimated using the standard deviation of the fitting: CA (*σ* = 271.9), CA + 10 eq. A8 (*σ* = 1104.1), CA + 10 eq. A8 + 50 eq. H1 (*σ* = 665.6). See Fig. S30–40† for full *m*/*z* scale MALDI analyses. (b) UV monitoring (linear range, 0–300 min) of the adaptive regulation proposed for the hydrolysis of *p*-NPA in the presence of CA, A8, and H1. Reaction conditions: D_2_O (pD = 7.2), 335 K. See Fig. S15† for the kinetic profile obtained in the presence of solely CA (1 μM) and H1 (10 μM). (c) Representation of the “turn on–off” hydrolase activity of *p*-NPA attained using A8 and H1 as dynamic effectors.

Since the bioconjugations were based on imine formation, we considered that the reversible covalent bonds could be displaced by the influence of an external molecule, offering a mechanism to revert the modification of CA by A8. One may presume that the introduction of an acylhydrazide can trigger a release of the dynamic residue through aldimine component exchange, as acylhydrazones present a higher thermodynamic stability than imines.^47^ Girard's reagent T (H1, Fig. 7b) was selected as the potential effector as it contains a positively charged moiety in its structure, which might favor the rearrangement process through attractive supramolecular interactions with the carboxylate group of A8. The kinetic profile for the formation of A8H1 from A8 and H1 was studied using ^1^H NMR spectroscopy (Fig. S14†). After 2 h of reaction, poor yields were obtained when the two reagents were mixed in a 1 : 1 molar ratio. On the other hand, the addition of 10 eq. of H1 led to yields higher than 50% in 30 min of reaction, with almost quantitative formation of the aldimine after 120 min (Fig. S14†). MALDI analysis of a sample containing CA, 10 eq. of A8, and 50 eq. of H1 showed a decrease in *m*/*z* in comparison with the value measured for the inhibited enzyme (see green and purple distributions in Fig. 7a, respectively). Therefore, acylhydrazide H1 seemed to be able to react with the bioconjugated units of A8 to form A8H1, releasing CA in its native state. This was in good accordance with the catalytic assays performed. Upon introduction of H1 (10 μM) in a solution containing the inhibited enzyme (1 μM CA + 1 μM A8), and equilibration for 2 h to assure the complete formation of acylhydrazone, the initial rates of *p*-NPA hydrolysis provided by native CA were attained (Fig. 7b). These results highlight the potential of using dynamic inhibitors based on reversible covalent bonds that permit an “on–off” bioconjugations to control sophisticated enzymatic processes (Fig. 7c).

## Conclusions and perspectives

Herein we showed that by exploiting supramolecular multivalent complementarity, one can efficiently drive imine formation even under physiological conditions. Specifically, combined electrostatic forces and hydrogen bonds between sidechains of amines and aldehydes can enhance the stability of the corresponding imines. This approach was further corroborated by simple experiments using model substrates, showing that in the cases where a supramolecular mismatch was introduced (*e.g.*, two building blocks containing negatively charged units), no aldimine product was observed. The best-performing aldehydes A7 and A8 among those studied here, were reacted with amino acid derivatives and small peptides, yielding excellent selectivities for the functionalization of the terminal Nα, even in substrates containing competing sites. The bioconjugation to carbonic anhydrase (CA) was also assayed for studying the aldimine formation in biomolecules with such high functional density, observing that up to four units of A8 were apparently bound to CA using 10 eq. of aldehyde. Spectroscopic analyses revealed that these modifications based on reversible covalent bonds were perturbing the 3D-structure of the protein, opening the way to studying their effect on the enzymatic activity. Remarkably, although A7 barely affected the catalytic performance of CA, the introduction of 10 eq. of A8 resulted in 93% inhibition of the initial enzymatic activity. Moreover, thanks to the reversible nature of the imine bonds, it was possible to design a “turn on–off” process in which the introduction of an effector, namely H1, led to the recovery of the native activity of CA. Such adaptive modifications of proteins showcase the enzymatically-reversible transformations that maintain the metabolic balance as a function of molecular environmental requirements. The implementation of the concept of supramolecular multivalency may become a major asset for improving the efficiencies of challenging reactions in highly competitive media.

## Data availability

The ESI† contains experimental details, characterization, spectral data, computational data and cartesian coordinates.

## Author contributions

The manuscript was written through contributions of all authors. All authors have given approval to the final version of the manuscript. Conceptualization: F. E., J. M. L. Data curation: F. E., F. R., formal analysis: F. E. Funding acquisition: J. M. L. Investigation: F. R. (supporting), F. E. (lead). Methodology: F. E. Project administration: J. M. L. Resources: J. M. L. Supervision: F. E., J. M. L. Validation: F. E. Visualization: F. R. (supporting), F. E. (lead), writing original draft: F. E. Writing – review and editing: F. R. (supporting), F. E., J. M. L.

## Conflicts of interest

There are no conflicts to declare.

## Supplementary Material

SC-014-D3SC04128J-s001
