# Supplementary material for: Supramolecular multivalency effects enhance imine formation in aqueous medium allowing for dynamic modification of enzymatic activity
Source: Chem Sci. 2023 Sep 7;14(37):10249–57. doi: 10.1039/d3sc04128j (PMC10530293; doi:10.1039/d3sc04128j)
Supplement: SC-014-D3SC04128J-s001 [file SC-014-D3SC04128J-s001.pdf]

Electronic Supplementary Material (ESI) for Chemical Science.
This journal is © The Royal Society of Chemistry 2023

# Electronic Supporting Information

## Supramolecular multivalency effects enhance imine formation in aqueous medium allowing for dynamic modification of enzymatic activity

Ferran Esteve,*[a] Fidan Rahmatova,[a] Jean-Marie Lehn*[a]

[a]*Laboratoire de Chimie Supramoléculaire, Institut de Science et d'Ingénierie Supramoléculaires (ISIS), Université de Strasbourg, 8 allée Gaspard Monge, 67000 Strasbourg, France*

## Table of contents

**1. Experimental section** ........ S1

**2. Main text supporting Figures and Schemes** ........ S5

**3. Characterization** ........ S13

**4. MALDI analyses** ........ S23

**5. DFT cartesian coordinates and energies** ........ S29

**6. References** ........ S42

## 1. Experimental section

### Materials and Methods

All amines, aldehydes and components of buffer solutions were obtained from commercial suppliers and used without further purification. Carbonic anhydrase from bovine erythrocytes (**CA**) was obtained from SigmaAldrich. No unexpected or unusually high safety hazards were encountered.

A Mettler Toledo SevenCompact pH Meter S220 was used to monitor the pH of the solutions, adjusted with either NaOD or DCl solutions as appropriate. All reactions were performed at room temperature, unless otherwise noted. Imination reactions were carried out in 50 mM deuterated phosphate buffer pD 7.2 unless otherwise indicated. The yields and product abundances were determined by relative integration of the signals in the 500 MHz $^{1}$H-NMR spectra (Bruker Ascend Spectroscope Advance Neo-500 MHz; 500 MHz for $^{1}$H and 125 MHz for $^{13}$C{$^{1}$H}). An example of the procedure is illustrated in Fig. S16 and S17 in the characterization section. First, the spectrum was referenced, phased, and the baseline was corrected. Afterwards, the signals of interest were accurately integrated to minimise the residual error, and finally, the numerical values are processed (*e.g.,* integration of the bisimino species is divided by two) for calculating product abundances. The error in integration is of 5%. The identification of the bisimine species was also confirmed by HRMS. All the reactions were analysed after 10 min, 1 h, 24 h, and 48 h to confirm that the equilibrium state was reached. The equilibrium was attained in all reactions after 10 min, except for the condensations between **T** and **A2**/**A3** in which 1 h was needed. Data analysis was performed using MestReNova (version 14.2.3) and OriginPro (version 9.8.0.200). HRMS-electro-spray ionization (HRMS-ESI) mass spectra were recorded using a ThermoFisher Exactive Plus EMR Orbitrap mass spectrometer. MALDI analyses were performed on a MALDI AUTOFLEX SPEED Bruker using para-Nitroaniline PNA as the matrix. UV-VIS spectroscopy measurements were performed on a JASCO V-670 UV-VIS spectrophotometer. CD spectroscopy measurements were performed on a J-1500 CD spectrophotometer.

### Catalytic experiments and CA inhibition

A method was properly designed for monitoring the kinetic profiles of the assayed hydrolysis reactions. All catalytic transformations were performed in $D_2O$ (pD 7.2, 50 mM phosphate buffer) at 335 K, and the concentrations of **p-NP** were determined using a calibration curve (absorbance at 400 nm). In a typical experiment (inhibition of **CA** using **A8** in a 1:1 molar ratio), 10 µL of a 0.1 mM solution of **CA** were diluted with 970 µL of $D_2O$ (pD = 7.2, PBS, 50 mM). To this solution, 10 µL of a 0.1 mM solution ($D_2O$, pD = 7.2, PBS, 50 mM) of **A8** were added and the mixture was equilibrated for 10 min. Subsequently, 10 µL of a 10 mM solution of **p-NPA** ($CH_3CN$) were added and the kinetic profile for the hydrolysis reaction was monitored over 20 min.

### Titration experiments

The concentration of **CA** was maintained constant during the consecutive additions of **A8**. For the NMR titration, 0.3 mM solution of **CA** in 0.4 mL of $D_2O$ (pD = 7.2, PBS, 50 mM) was treated with increasing amounts of a 1 mL stock solution (pD = 7.2, PBS, 50 mM) of **A8** (10 mM) that also contained 0.3 mM of **CA.** A similar procedure was followed for the CD titration experiment, using

in this case a 0.001 mM solution of **CA** and a 0.1 mM stock solution of **A8** (containing 0.001 mM of **CA**), both in $D_2O$ (pD = 7.2, PBS, 50 mM).

**DFT calculations**

DFT calculations were performed using Gaussian 09 (revision B.01) at the b3lyp level of theory using the 6-31g(d,p) basis set.[1,2] All free energies (kcal/mol) were calculated taking into account the energies of the corresponding reagents and the molecule of water (as its $H_3O^+$ ion, considering that the amino components were calculated as their ammonium derivatives) released in the condensation reaction. The DFT models were calculated at room temperature and using water as the solvent (PCM).[3] 3D representations were designed using the Mercury software. It must be noted that the free-energy values are only suitable for comparison between systems, as the energy of the compounds will depend on the protonation degree of each of the species, and thus it might change according to both pKa of the corresponding components and the pH of the medium. For all calculations, the highest degree of protonation was considered for the compounds to maximize the effect of possible interactions between charged groups.

**Docking analyses**

SwissDock is an open source web server-based molecular docking engine, and it can be accessed at http://www.swissdock.ch/. This webserver is developed and maintained by the Swiss Institute of Bioinformatics, Lausanne, Switzerland.[4] The molecular structure of **CA** was download from PDB database (pdb: 1v9e). The molecular structure of **A8** was modelized at the b3lyp level of theory using the 6-31g(d,p) basis set. Both structures were uploaded on the SwissDock web server, and their interaction was elucidated using a blind and accurate docking for screening all possible recognition sites. Binding energies for the different interaction modes were recorded. The top-ranked pose was selected ($\Delta G$ = -8.15 kcal/mol) and it was represented using Chimera 1.16 software (Fig. S10).

**Synthetic protocols**

**Synthesis of A9.** $K_2CO_3$ (1.2 eq., 0.67 g, 4.86 mmol) was suspended on dry DMF (10 ml) in an atmosphere of $N_2$. 3-thiolpropanoic acid (1 eq., 0.43 g, 0.35 mL, 4.05 mmol) was then dissolved in DMF (10 mL) and added to the reaction suspension. 2,3,4,5,6-Pentafluorobenzaldehyde (**A6**) (1 eq., 0.79 g, 0.5 ml, 4.05 mmol) was introduced to the mixture and stirred for 5 h at 20°C. The solvent was removed in vacuo and acid water (pH 4, 20 ml) was added to the dried solid. The resulting solution was extracted with EtOAc (3x40 mL). The combined organic phases were dried over $Na_2SO_4$, filtered, and evaporated under reduced pressure to yield pure **A6** (449 mg, 1.59 mmol, 39% yield) as a yellow solid. Characterization: m.p.= 103-105 ºC. FTIR (ATR): 2899, 2706-2498(bs), 1692, 1636, 1466, 1409, 1279. $^1$H-NMR (500 MHz, DMSO-$d_6$) δ (ppm) 12.42 (s, 1H), 10.16 (s, 1H), 3.24 (t, J = 6.79 Hz, 2H), 2.58 (t, J = 6.79 Hz, 2H). $^{13}$C{$^1$H}-NMR (500 MHz, DMSO-$d_6$) δ (ppm) 184.0, 172.8, 147.4, 145.6, 121.3, 114.8, 35.1, 29.8. HRMS-QTOF(-) calcd for $C_{10}H_5F_4O_3S$: 280.9901, found 280.9890. See Fig. S18-S20 and S27 for characterization.

**Synthesis of A8.** This compound was synthesized following the same procedure as described for **A6** (0.42 g, 0.98 mmol, 21% yield, brownish solid). Characterization: m.p.= 191-192 ºC. FTIR (ATR): 3311, 2959, 2802-2408(bs), 1746, 1702, 1635, 1509, 1278, 1212. $^{1}$H-NMR (500 MHz, DMSO-d$_6$) δ (ppm) 13.07 (s, 1H), 10.13 (s, 1H), 7.71 (d, J = 8.6 Hz, 1H), 7.37-7.28 (m, 6H), 4.98 (s, 2H), 4.18 (m, 1H), 3.58-3.51 (m, 2H). $^{13}$C{$^{1}$H}-NMR (500 MHz, DMSO-d$_6$) δ (ppm) 183.9, 171.8, 156.3, 147.5, 145.4, 136.7, 128.8, 128.4, 128.0, 121.2, 114.8, 66.1, 54.9, 35.2. HRMS-QTOF(-) calcd for $C_{18}H_{12}F_4O_5SN$: 430.0378, found 430.0367. See Fig. S21-S23 and S28 for characterization.

**Synthesis of A7.** This compound was synthesized following the same procedure as described for **A6** (0.88 g, 2.703 mmol, 53% yield, yellow solid). Characterization: m.p.= 157-158 ºC. FTIR (ATR): 2873, 2702-2525(bs), 1698, 1635, 1465, 1420, 1278. $^{1}$H-NMR (500 MHz, DMSO-d$_6$) δ (ppm) 12.88 (bs, 2H), 10.16 (s, 1H), 4.10 (dd, J = 7.78, 6.56 Hz, 1H), 2.78 – 2.71 (m, 2H). $^{13}$C{$^{1}$H}-NMR (500 MHz, DMSO-d$_6$) δ (ppm) 183.9, 171.7, 171.3, 148.6, 146.6, 117.9, 116.5, 45.3, 36.6. HRMS-QTOF(-) calcd for $C_{11}H_5F_4O_5S$: 324.9799, found 324.9788. See Fig. S24-S26 and S29 for characterization.

## 2. Main text supporting Figures, Schemes and Tables

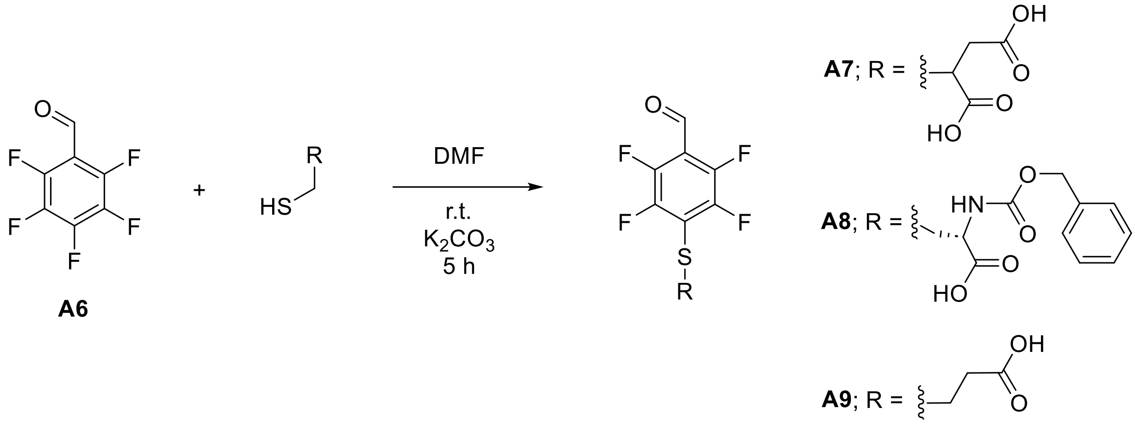


**Scheme S1.** Synthetic route for the herein proposed water-soluble reactive aldehydes, *viz.* **A7**, **A8**, and **A9**.

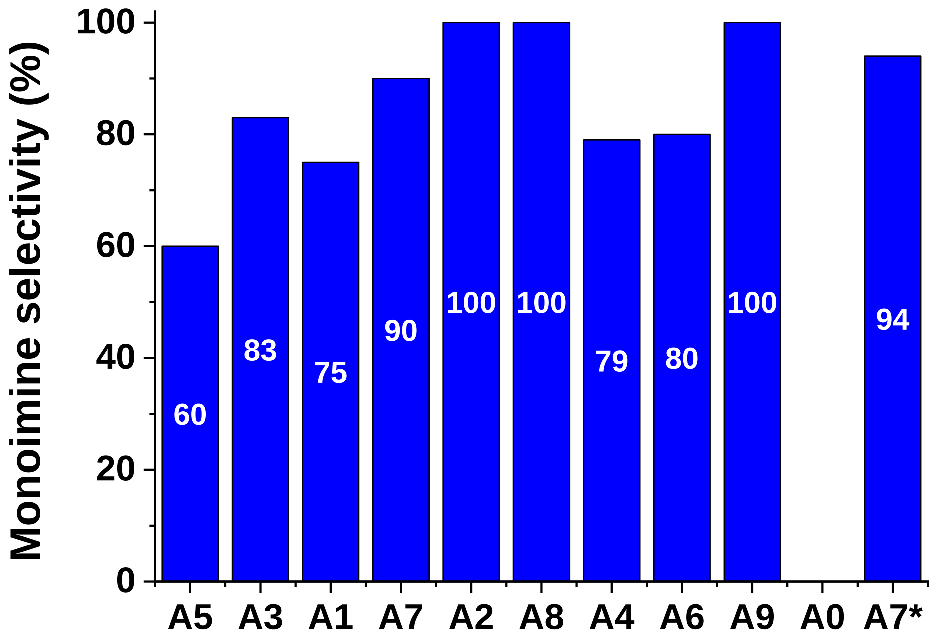


**Fig. S1.** Monoimine selectivity (over the bisimino species detected) attained in the reaction between **T** and the different aldehydes assayed. Selectivity calculated according to the formula: $S=100*I_{monoimine}/(I_{monoimine}+[I_{bisimine}/2])$. Integrations (I) determined using $^{1}$H NMR spectroscopy (500 MHz, 295 K). Reaction conditions: $D_2O$ (pD = 7.2, phosphate buffer 50 mM), 295 K, [**AX**] = [**T**] = 5 mM. A7*: [**A7**] = 5 mM, [**T**] = 50 mM. Results have been sorted following the order of Fig. 2 in the main text for clarity.

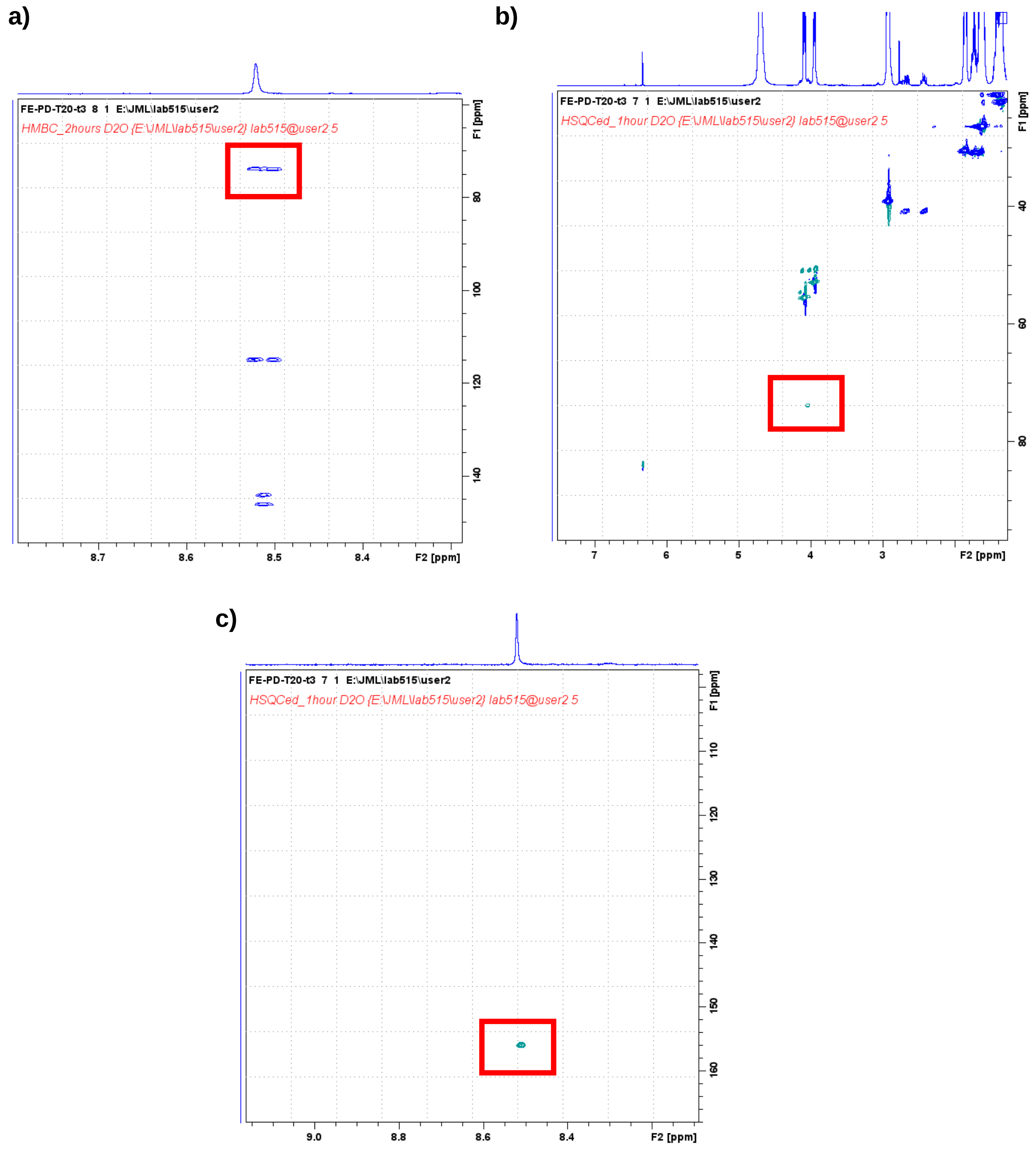


**Fig. S2.** 2D NMR analyses (500 MHz, 295 K, $D_2O$, pD = 7.2) for the reaction between **A7** and **KOMe** after 1 h. a) Partial HMBC showing the correlation between CH imine (8.52 ppm) and the $^{13}C$ signal at 73.7 assigned to the stereogenic carbon of **KOMe** (right next to the Nα). b) and c) show the partial edited HSQC in which the CH groups are coloured in green and the $CH_2$ in blue. The most relevant peaks have been highlighted with a red square for clarity.

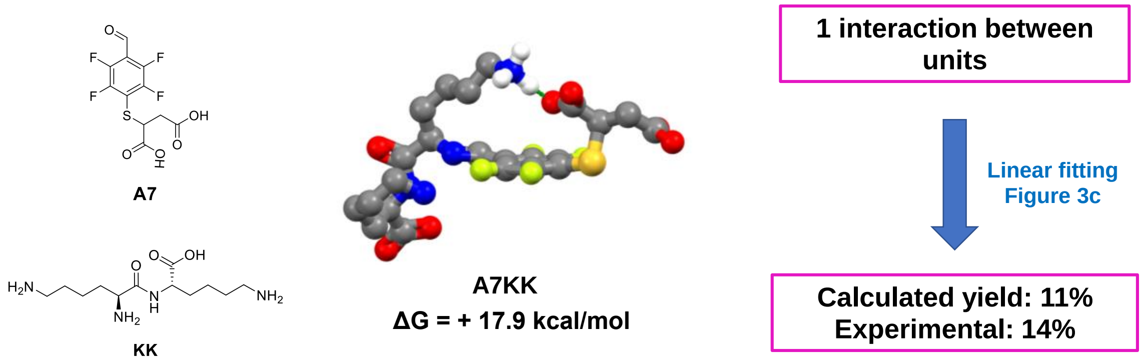


**Fig. S3.** DFT (b3lyp, 6-31g(d,p) calculated lowest in energy conformation for imine **A7KK**. The supramolecular interaction (hydrogen bond/electrostatic) has been highlighted with a discontinuous green line. Non-essential hydrogen atoms have been omitted for clarity.

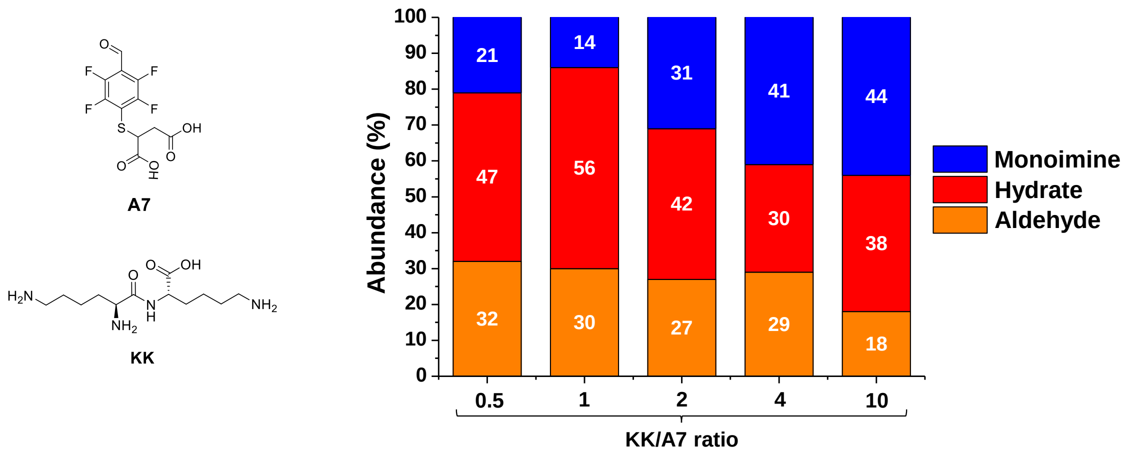


**Fig. S4.** Results for the **A7**:**KK** molar ratio assays. Component abundance (%) as calculated from $^{1}H$ NMR spectra (500 MHz, 295 K, $D_2O$). Reaction conditions: $D_2O$ (pD = 7.2, phosphate buffer 50 mM), 295 K, 1 h, 5 mM concentration for the limiting reagent.

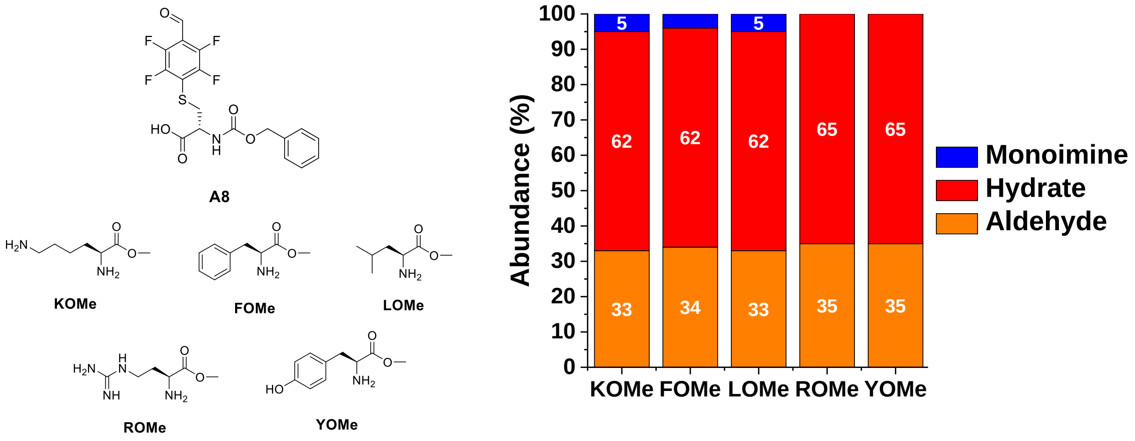


**Fig. S5.** Chemical structures of the aldehyde **A8** and the different methyl ester derivatives of selected amino acids. Component abundances (%) as calculated from $^{1}$H NMR spectra (500 MHz, 295 K, $D_2O$). Reaction conditions: $D_2O$ (pD = 7.2, phosphate buffer 50 mM), 295 K, 1h, [**A8**] = [**XOMe**] = 5 mM.

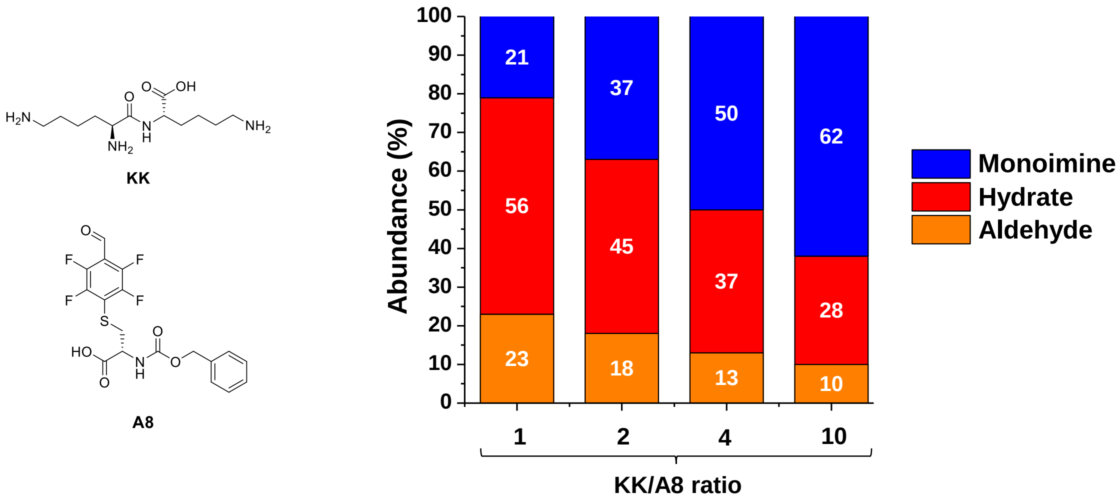


**Fig. S6.** Results for the **A8**:**KK** molar ratio assays. Component abundance (%) as calculated from $^{1}$H NMR spectra (500 MHz, 295 K, $D_2O$). Reaction conditions: $D_2O$ (pD = 7.2, phosphate buffer 50 mM), 295 K, 1 h, 5 mM concentration for the limiting reagent.

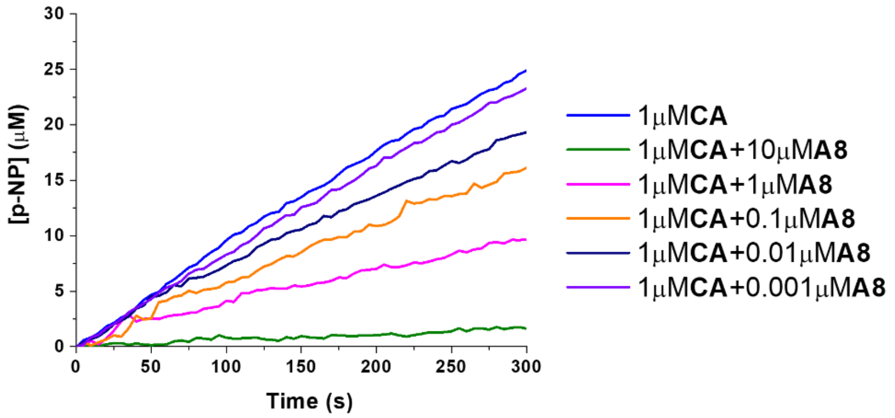


**Fig. S7.** UV monitoring (linear range, 0-300 min) of the hydrolysis of **p-NPA** in the presence of **CA** and different equivalents of **A8**. Reaction conditions: $D_2O$ (pD = 7.2), 335 K.

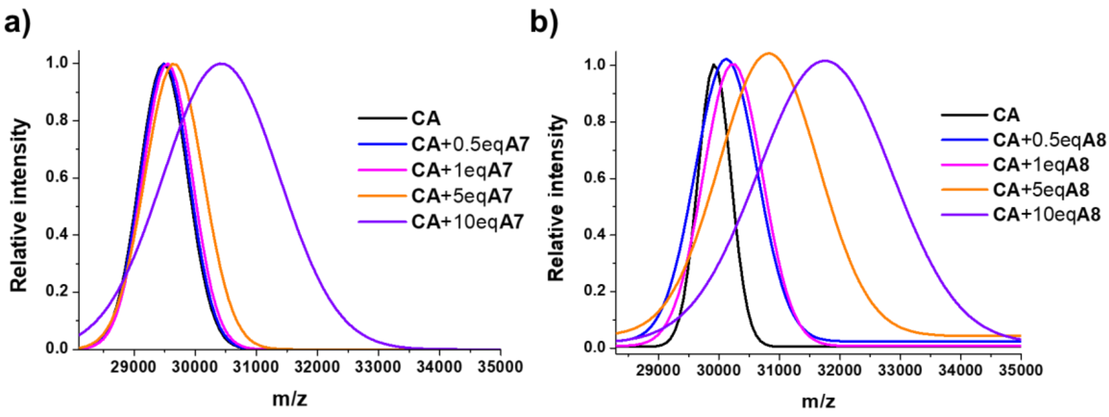


**Fig. S8.** MALDI analyses (PNA matrix, $D_2O$, pD 7.2 phosphate buffer) for **CA** (0.1 mM) with increasing amounts of **A7** (a) and **A8** (b). The peaks were fitted to a Gaussian distribution and the mean m/z was used for quantification of the bioconjugation efficiency; see Fig. 6e. See also Fig. SX-SY for full m/z scale MALDI analyses. The dispersity of the bioconjugation was estimated using the standard deviation of the fitting: (a) **CA** (σ= 395.4), **CA**+0.5eq**A7** (σ= 398.7), **CA**+1eq**A7** (σ= 407.3), **CA**+5eq**A7** (σ= 477.9), **CA**+10eq**A7** (σ= 950.3); (b) **CA** (σ= 271.9), **CA**+0.5eq**A8** (σ= 511.8), **CA**+1eq**A8** (σ= 488.3), **CA**+5eq**A8** (σ= 823.6), **CA**+10eq**A8** (σ= 1104.1).

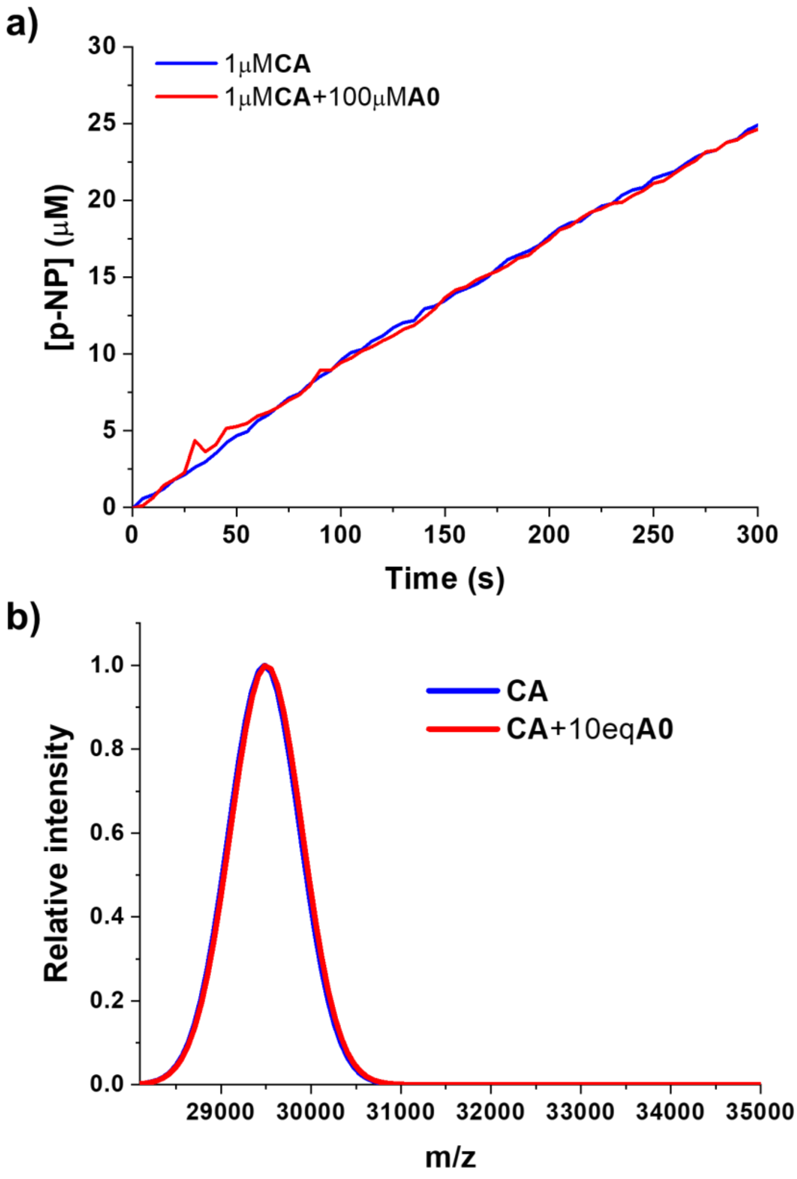


**Fig. S9.** (a) UV monitoring (linear range, 0-300 min) of the hydrolysis of **p-NPA** in the presence of **CA** and **A0** (100eq.). Reaction conditions: $D_2O$ (pD = 7.2), 335 K. (b) MALDI analyses (PNA matrix, $D_2O$, pD 7.2 phosphate buffer) for **CA** (0.1 mM) in the presence of **A0** (10eq.). The peaks were fitted to a Gaussian distribution.

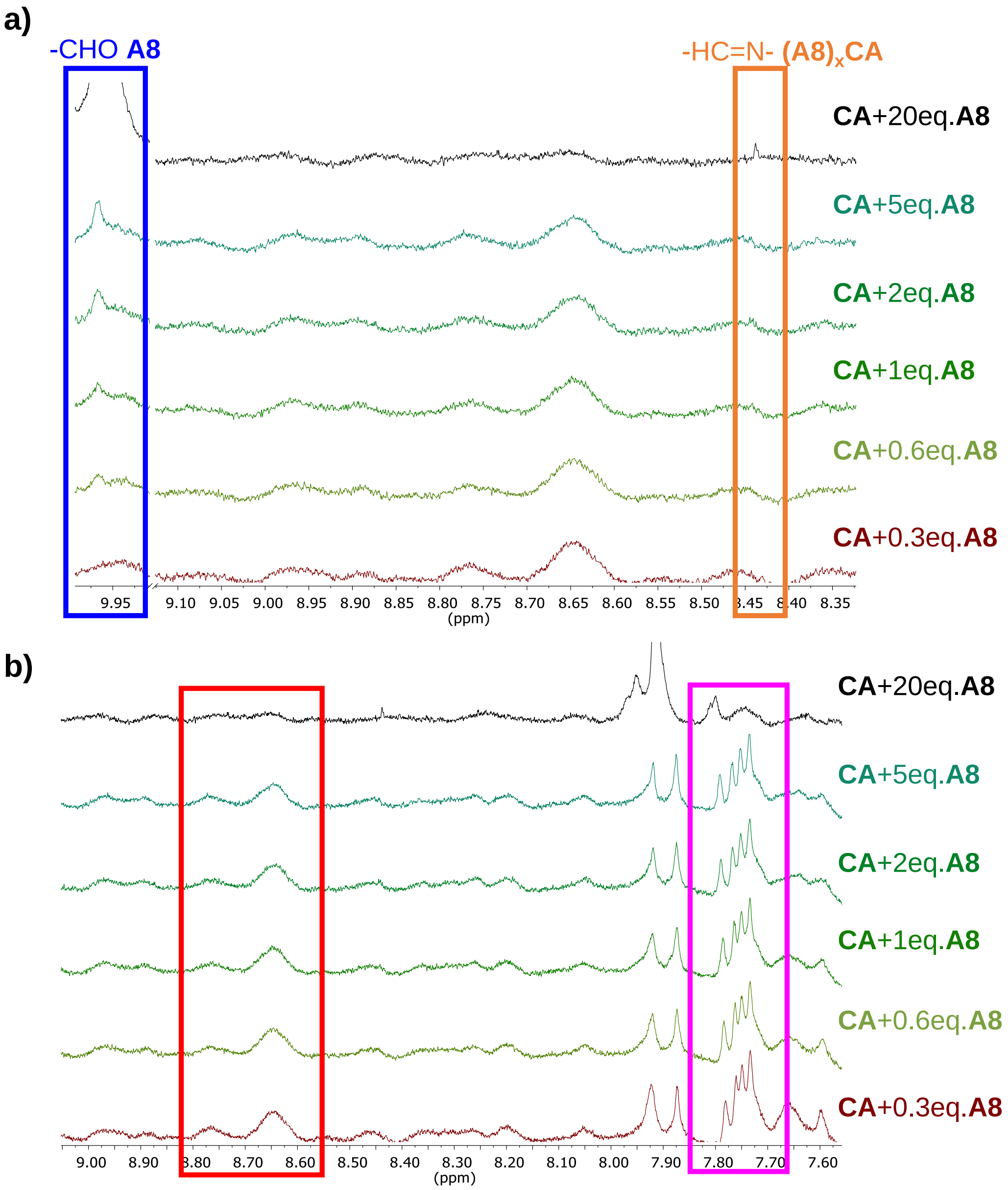


**Fig. S10.** Partial $^{1}H$ NMR (500 MHz, $D_2O$, pH = 7.2, 295 K) spectra for the titration experiment between **CA** (0.25 mM) and **A8**. a) Signals corresponding to aldehyde -CHO and imine -HC=N- have been highlighted with blue and orange rectangles, respectively. b) Regions of the spectra that experience a shift in the presence of **A8** have been highlighted with a pink rectangle. On the other hand, some regions of the spectra that do not experience such shifts in the presence of **A8** have been highlighted with a red rectangle.

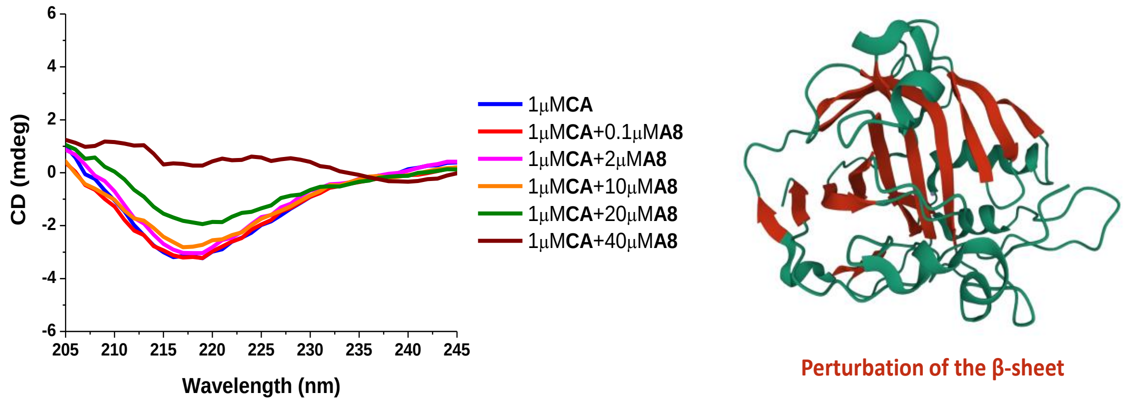


**Fig. S11.** Left: CD spectra ($D_2O$, pD = 7.2, phosphate buffer, 295 K) for the titration experiment between **CA** and **A8**. Right: 3D-structure of native **CA** from bovine erythrocytes (pdb: 1v9e). The backbone of the protein is coloured green and the β-sheet-like structures are highlighted in orange.

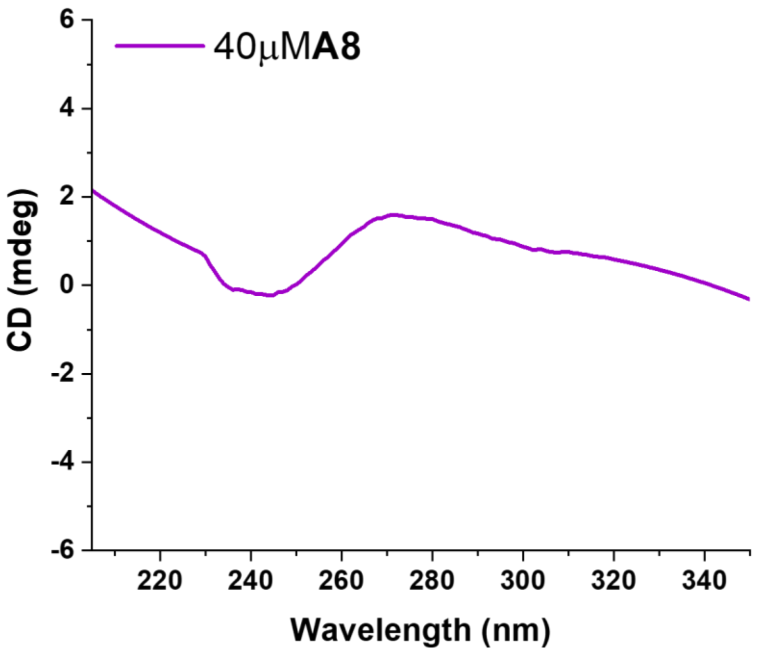


**Fig. S12.** CD spectrum ($D_2O$, pD = 7.2, phosphate buffer, 295 K) of a 40 μM solution in **A8**.

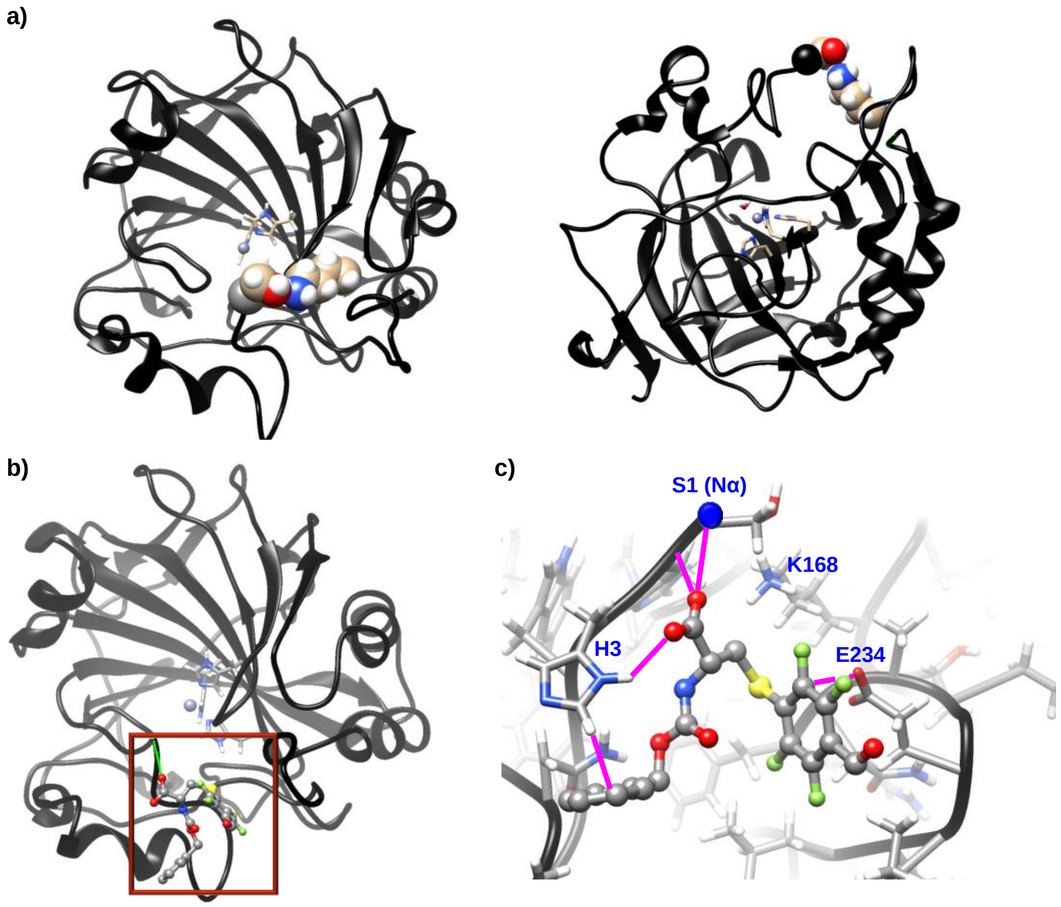


**Fig. S13.** a) Top (left) and side views (right) of **CA** (pdb: 1v9e). The backbone has been represented in black. The active site (in the centre of the conical cavity defined by β-sheets) has been highlighted in capped sticks ($Zn^{2+}$ ion represented with the spacefill model in grey). The Ser1 and K168 residues in the entrance of the cavity are highlighted using the spacefill model. b) Best docked pose for the **A8·CA** supramolecular adduct obtained using SwissDock. The region where **A8** is located has been highlighted with a dark red square. Binding free-energy: -8.15 kcal/mol. c) Zoomed-in region for the lowest in energy conformation obtained in the docking experiments. Supramolecular forces have been highlighted with pink lines. Compound **A8** has been represented using the ball and sticks model. All the representation were created using Chimera 1.16 software.

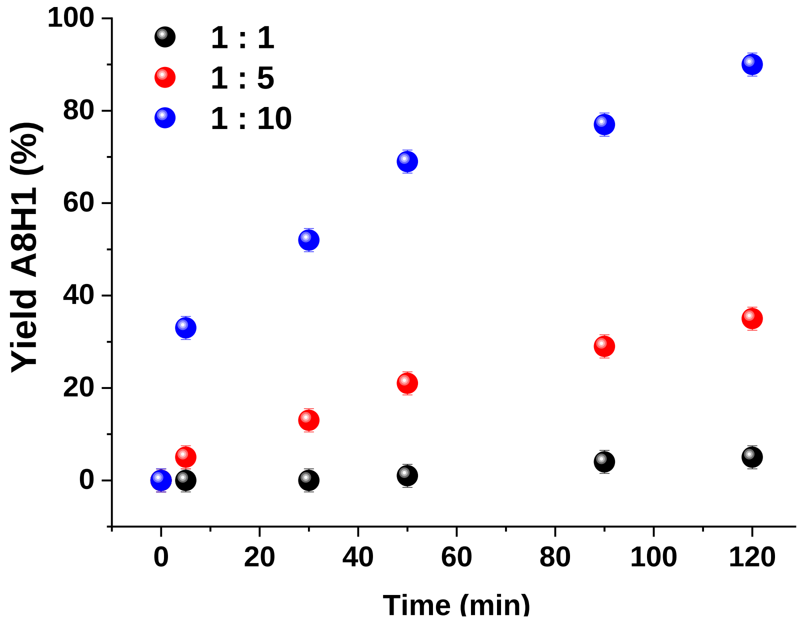


**Fig. S14.** Kinetic profiles for the reaction between **H1** and **A8**, as determined by $^{1}$H NMR spectroscopy (500 MHz, 295 K, $D_2O$, pD = 7.2). Concentrations: 5 mM each (black); 5 mM **A8** + 25 mM **H1** (red); 5 mM **A8** + 50 mM **H1** (blue). Error bars correspond to 5%.

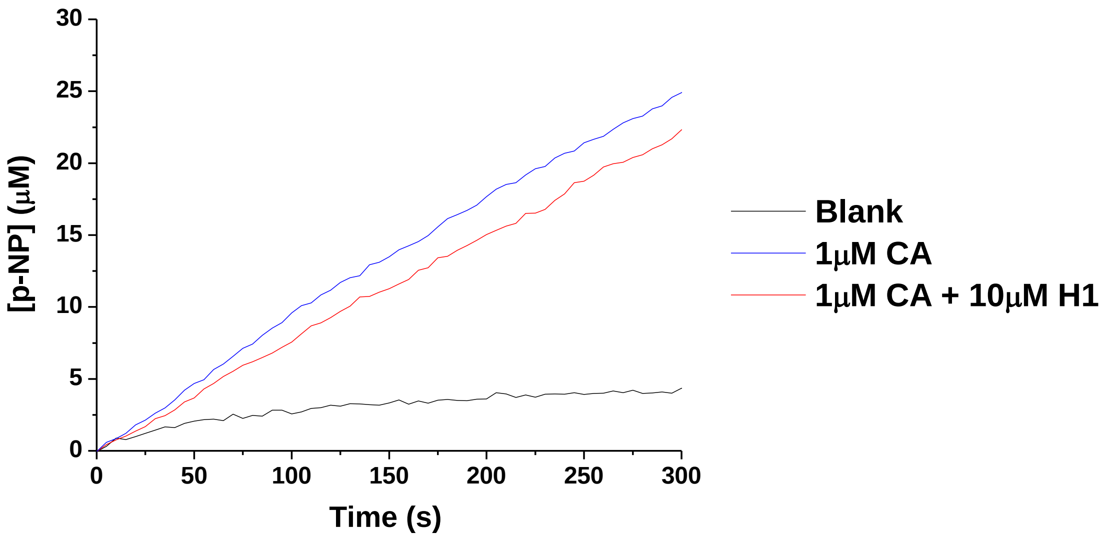


**Fig. S15.** UV monitoring (linear range, 0-300 min) for the hydrolysis of **p-NPA** in the presence of **CA**, and a **CA** : **H1** (1 : 10) mixture. Reaction conditions: $D_2O$ (pD = 7.2), 335 K. The blank has also been added for comparison.

## 3. Characterization

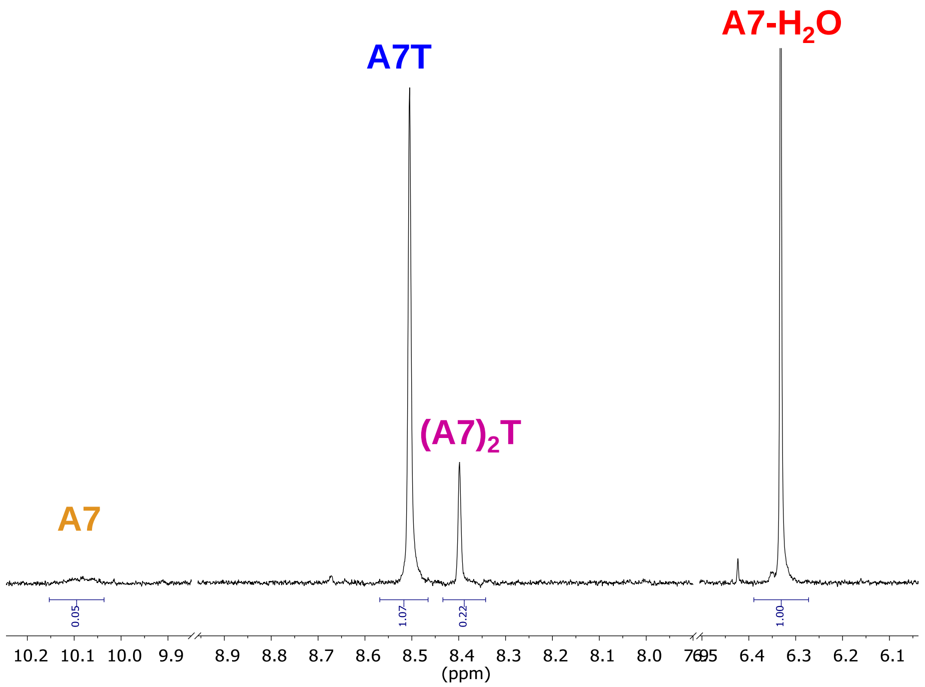


**Fig. S16.** Selected example for the product abundance quantification using $^{1}$H-NMR spectrum (500 MHz, $D_2O$ + phosphate buffer 50mM, pD 7.2, 295 K). The crude corresponds to the condensation reaction between **A7** and **T** (5 mM each) after 10 mins of equilibration. It must be noted that the integration of the **(A7)$_2$T** imine CH signal (8.4 ppm) was divided by two for the quantification of this species since there are two equivalent imine protons per molecule.

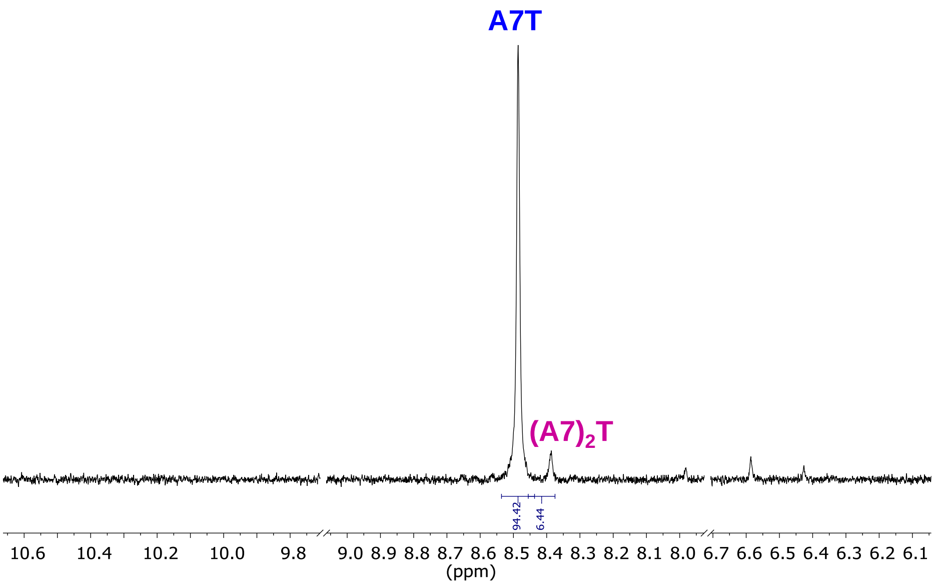


**Fig. S17.** Selected example for the product abundance quantification using $^{1}$H-NMR spectrum (500 MHz, $D_2O$ + phosphate buffer 50mM, pD 7.2, 295 K). The crude corresponds to the condensation reaction between **A7** and **T** (5 mM **A7** and 50 mM **T**) after 10 mins of equilibration. It must be noted that the integration of the **(A7)$_2$T** imine CH signal (8.4 ppm) was divided by two for the quantification of this species since there are two equivalent iminr protons per molecule.

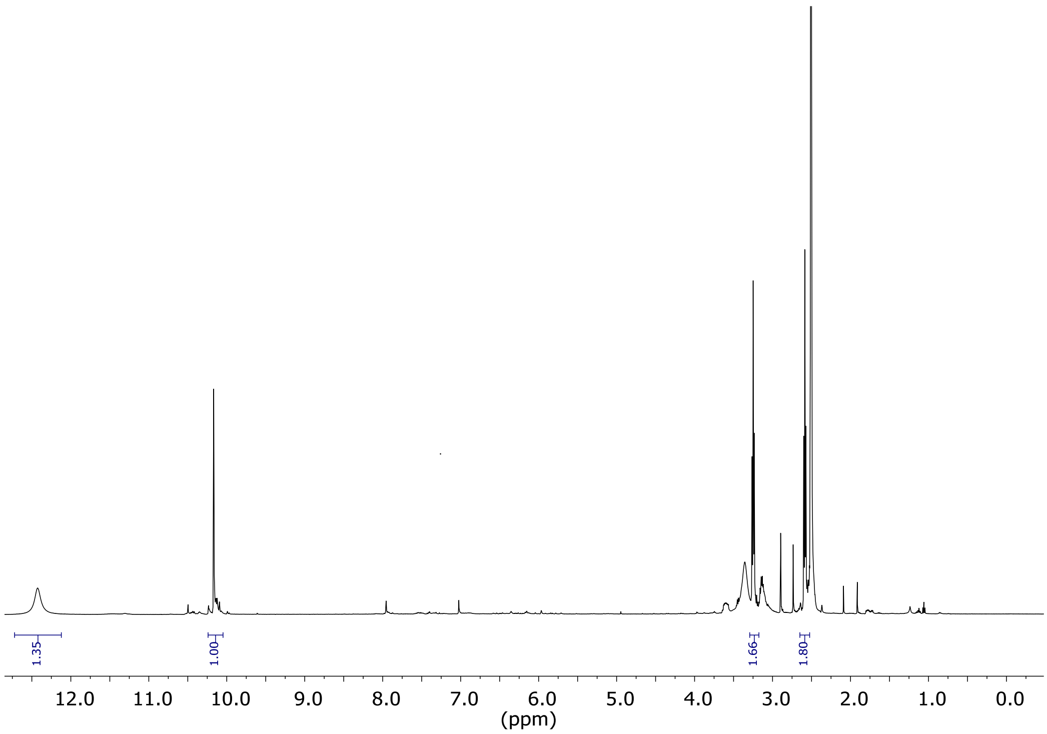


**Fig. S18.** $^{1}$H-NMR spectrum (500 MHz, DMSO-d$_6$, 295 K) of **A9**.

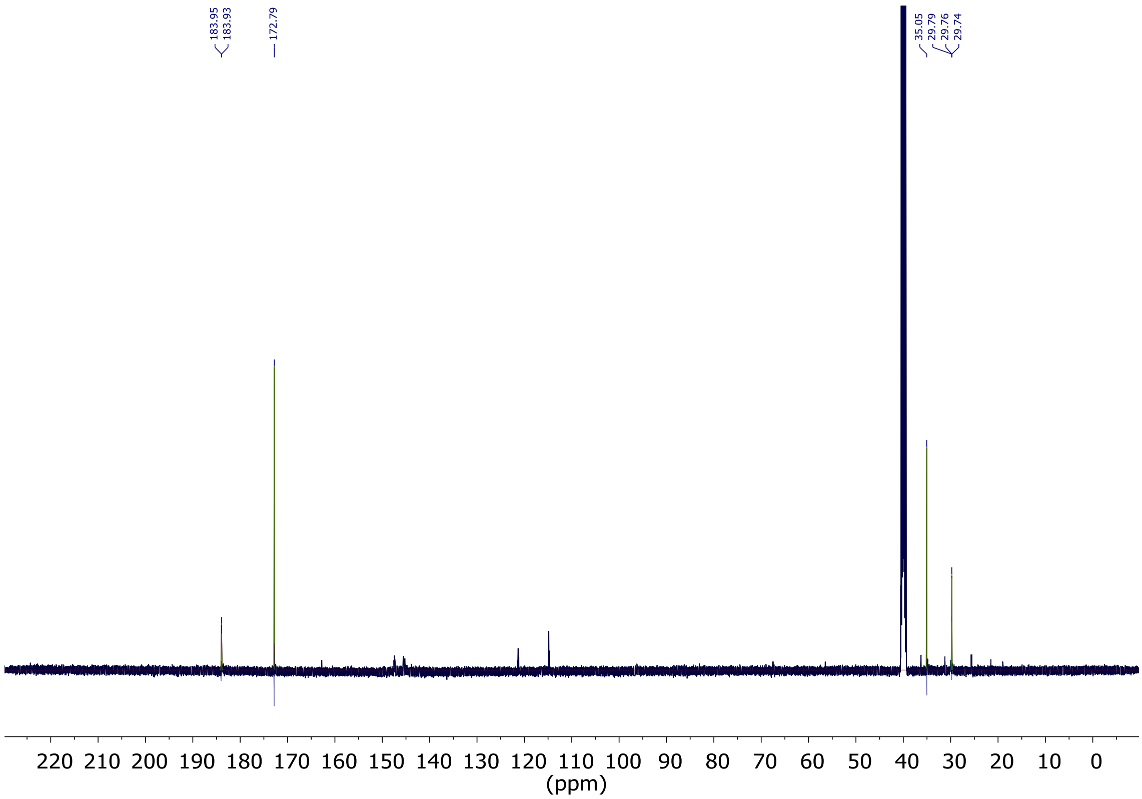


**Fig. S19.** $^{13}$C{$^{1}$H}-NMR spectrum (125 MHz, DMSO-d$_6$, 295 K) of **A9**.

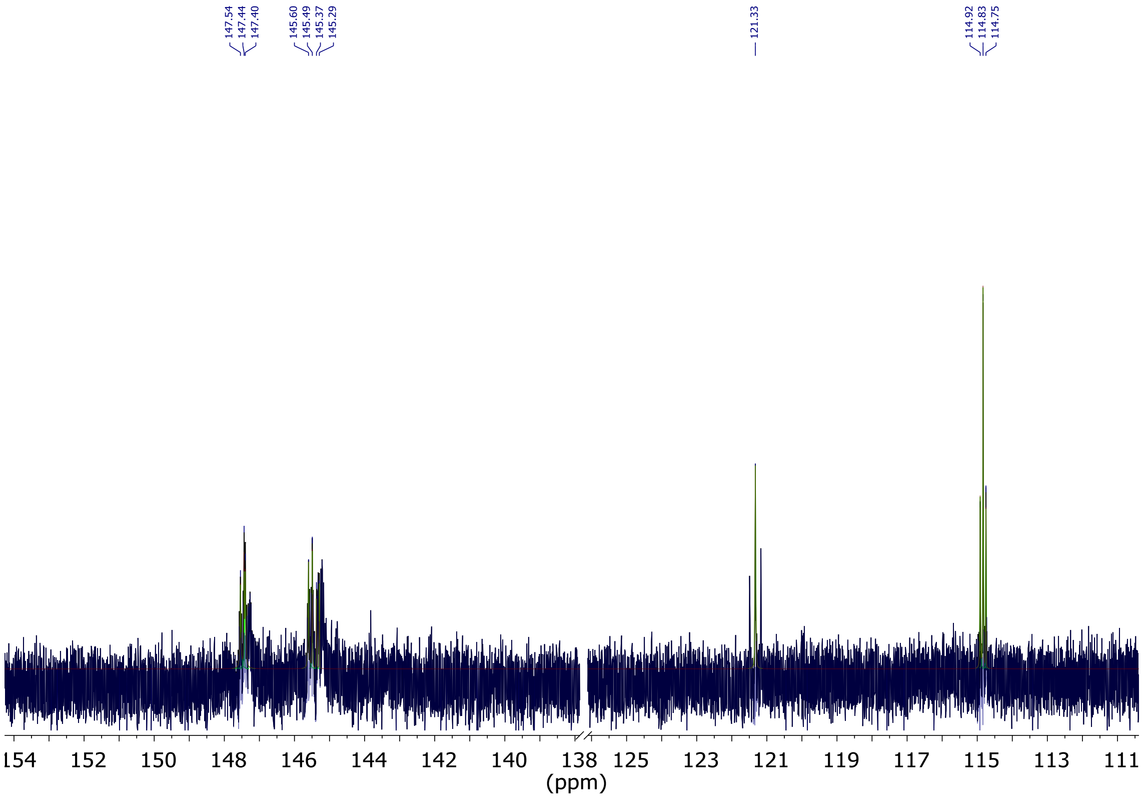


**Fig. S20.** Partial $^{13}C\{^1H\}$-NMR spectrum (125 MHz, DMSO-$d_6$, 295 K) of **A9**.

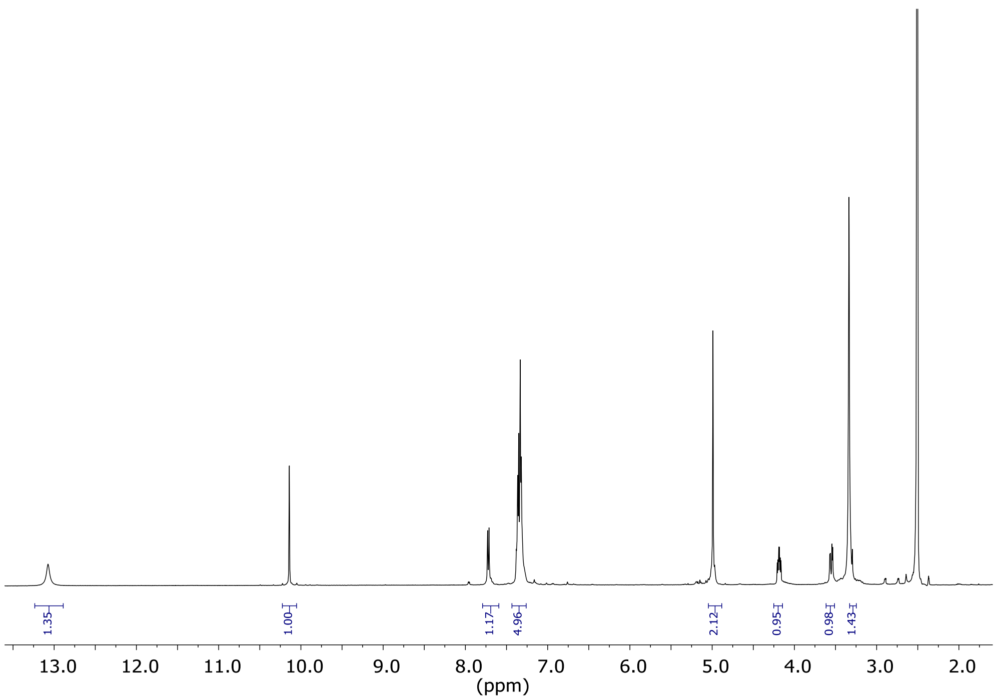


**Fig. S21.** $^{1}$H-NMR spectrum (500 MHz, DMSO-d$_6$, 295 K) of **A8**.

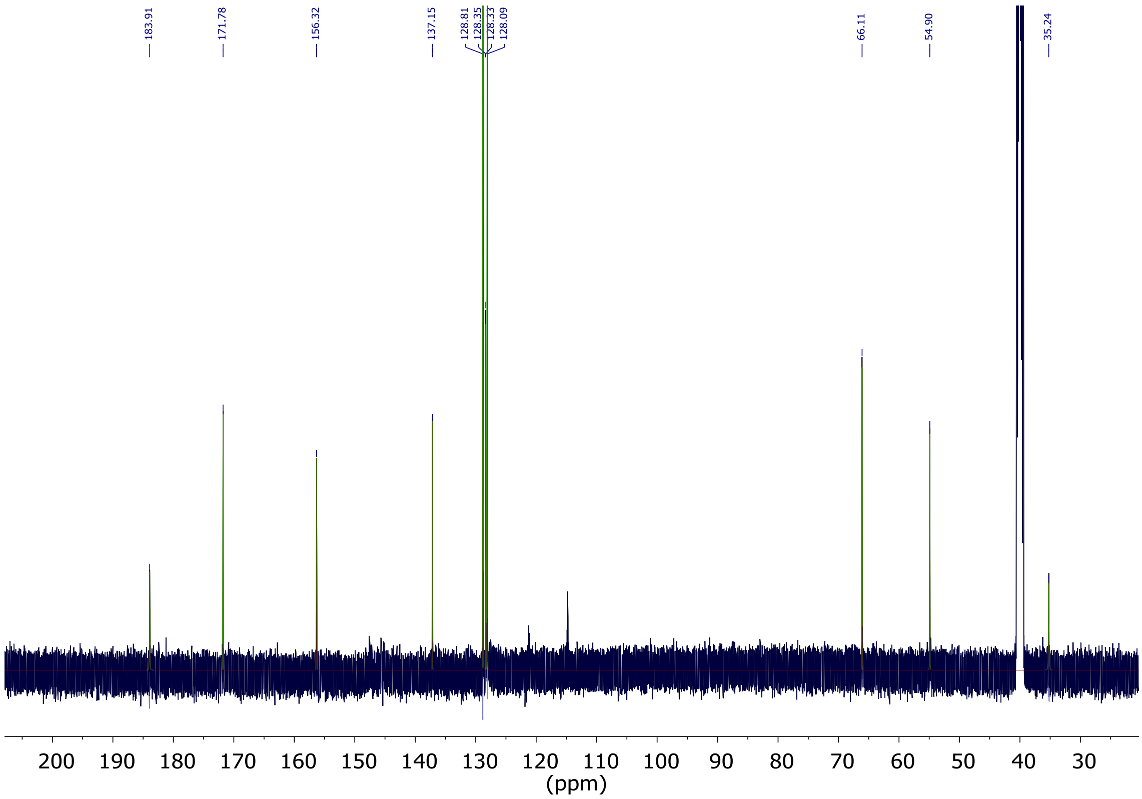


**Fig. S22.** $^{13}$C{$^{1}$H}-NMR spectrum (125 MHz, DMSO-d$_6$, 295 K) of **A8**.

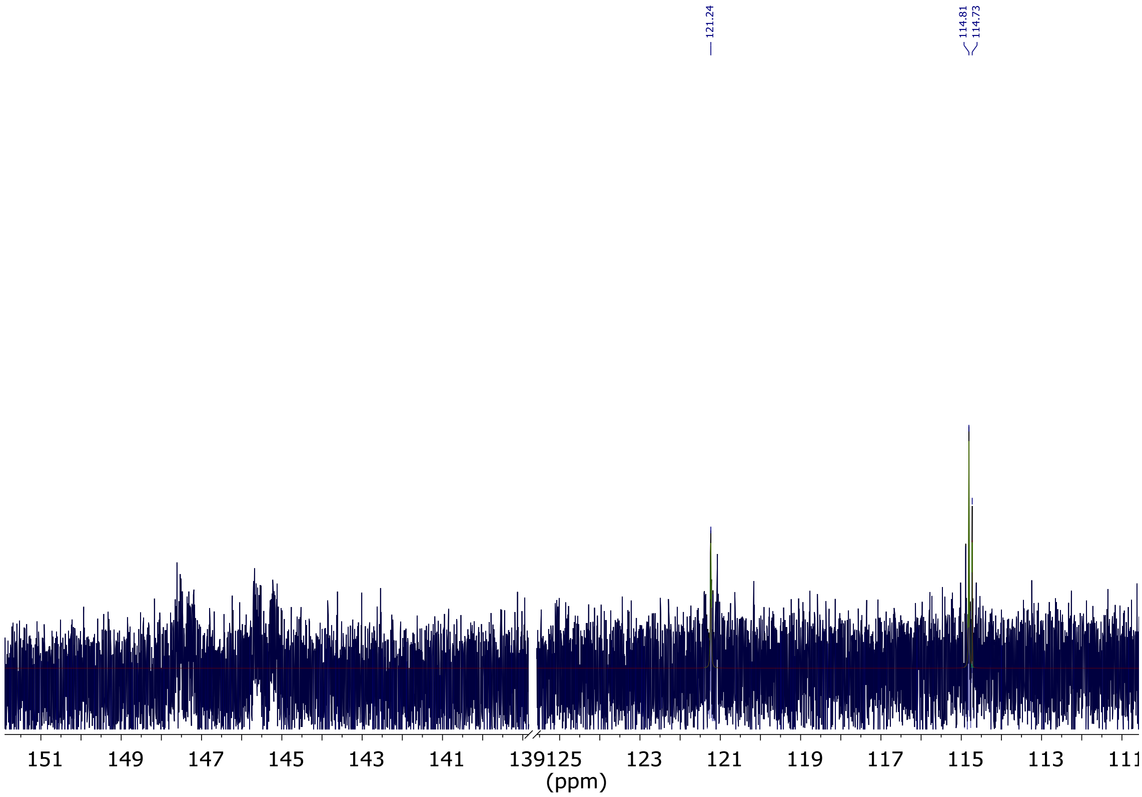


**Fig. S23.** Partial $^{13}C\{^{1}H\}$-NMR spectrum (125 MHz, DMSO-d$_6$, 295 K) of **A8**.

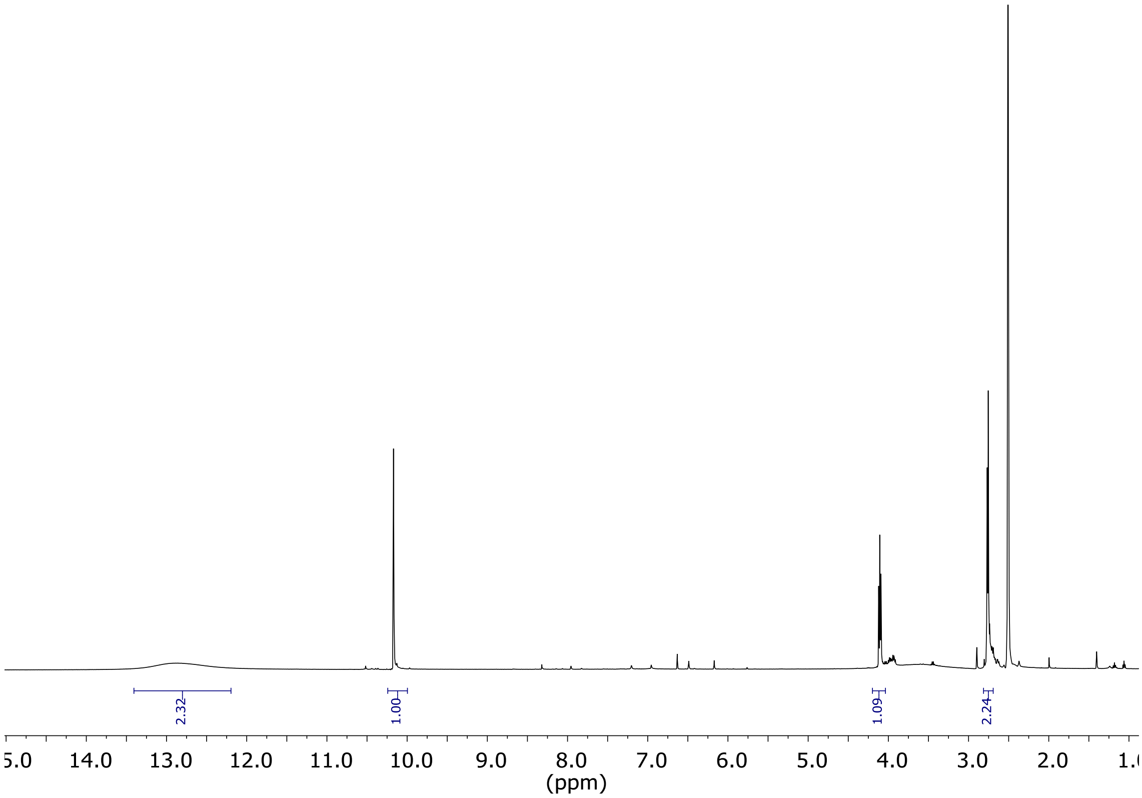


**Fig. S24.** $^{1}H$-NMR spectrum (500 MHz, DMSO-d$_6$, 295 K) of **A7**.

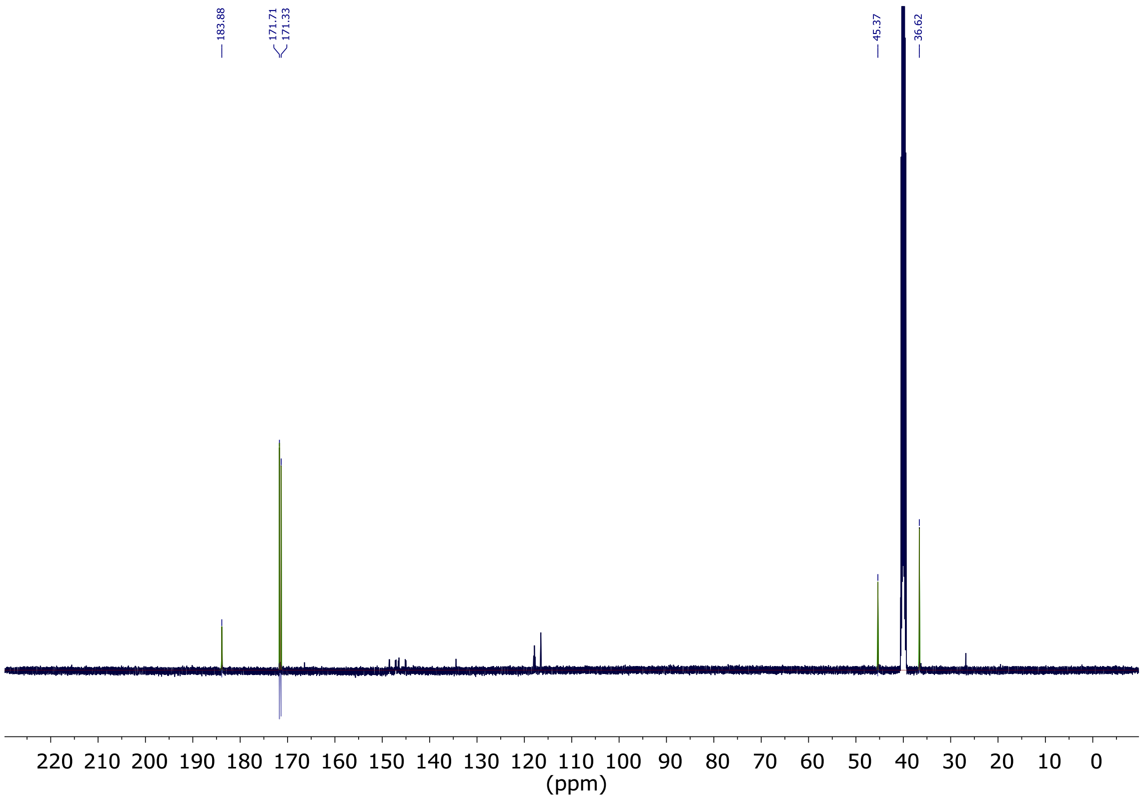


**Fig. S25.** $^{13}C\{^{1}H\}$-NMR spectrum (125 MHz, DMSO-d6, 295 K) of **A7**.

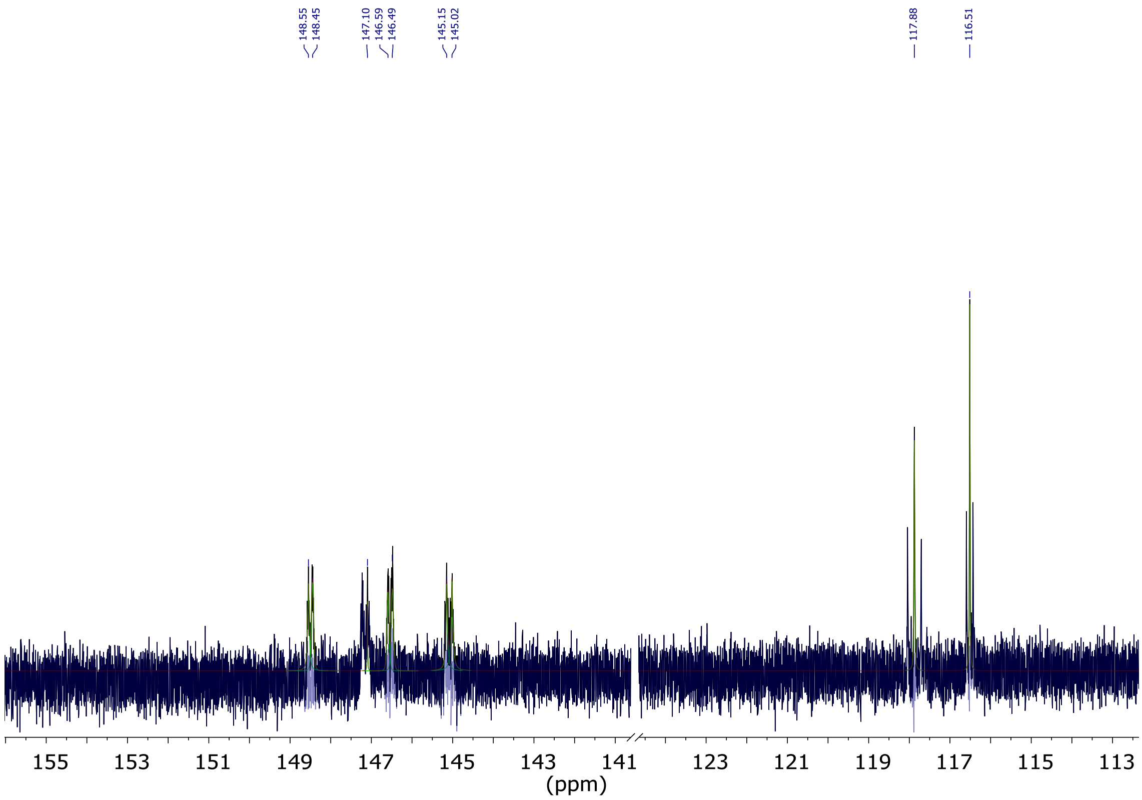


**Fig. S26.** Partial $^{13}C\{^{1}H\}$-NMR spectrum (125 MHz, DMSO-d6, 295 K) of **A7**.

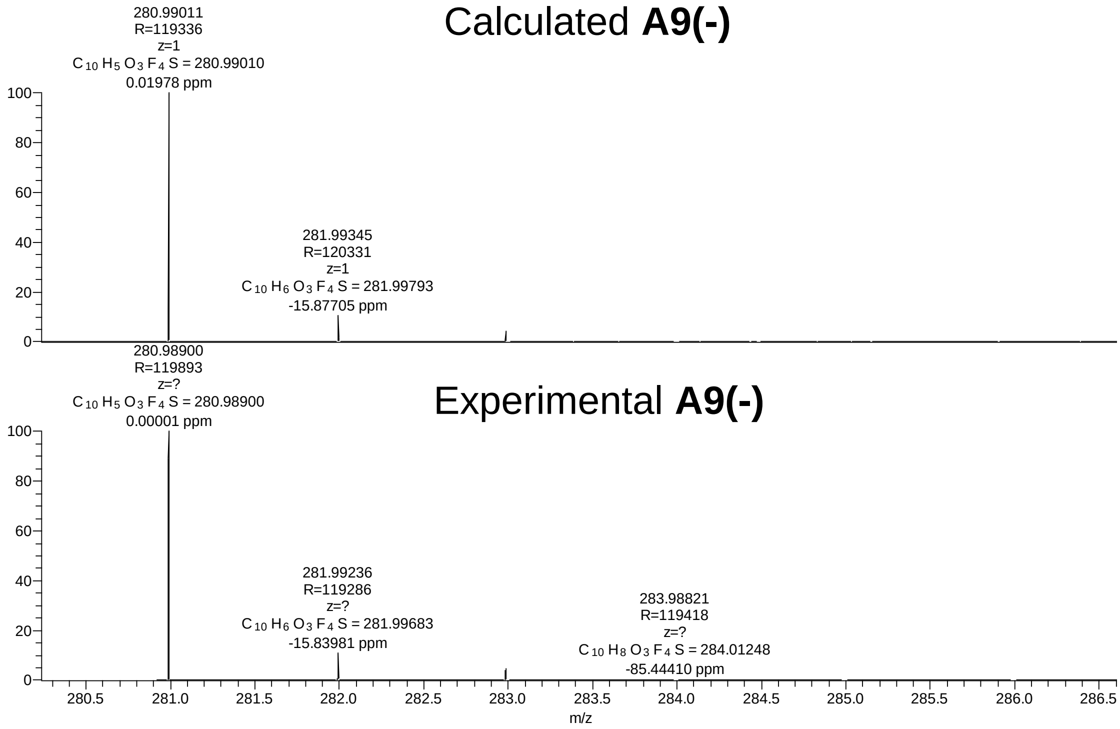


**Fig. S27.** HRMS-QTOF(-) spectrum (MeOH) of **A9**.

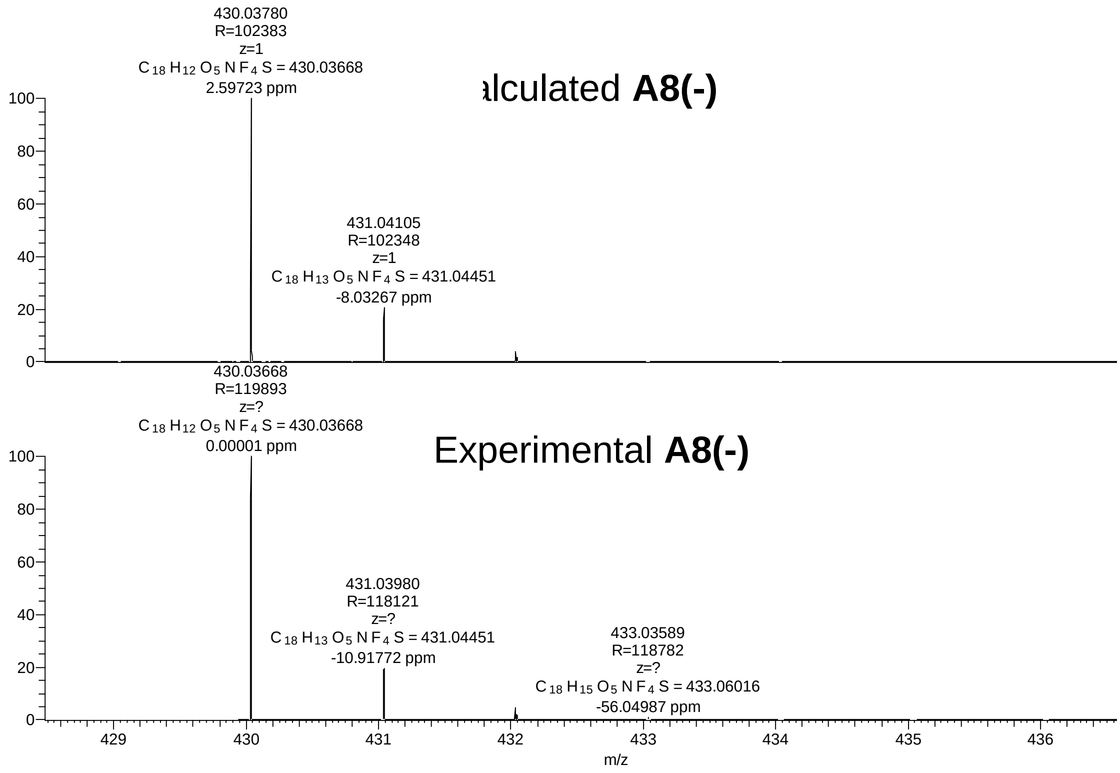


**Fig. S28.** HRMS-QTOF(-) spectrum (MeOH) of **A8**.

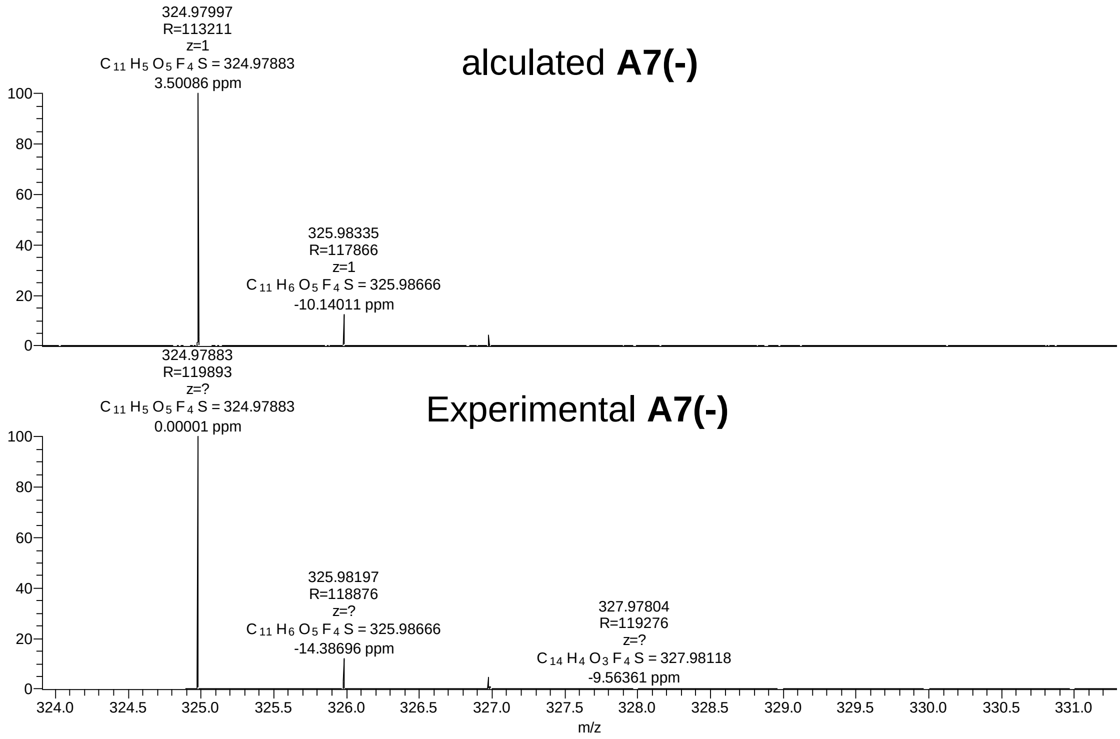


**Fig. S29.** HRMS-QTOF(-) spectrum (MeOH) of **A7**.

## 4. MALDI analyses

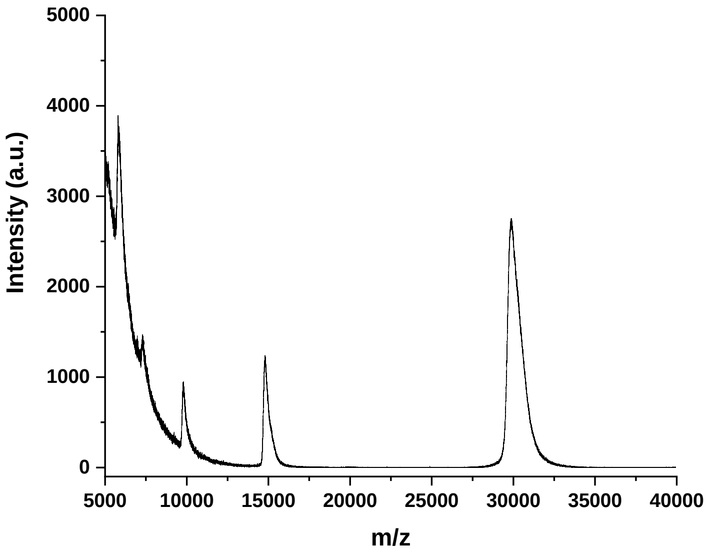


**Fig. S30.** MALDI analysis (PNA matrix, $D_2O$, pD 7.2 phosphate buffer) of **CA** (0.1 mM) in the absence of **A8**.

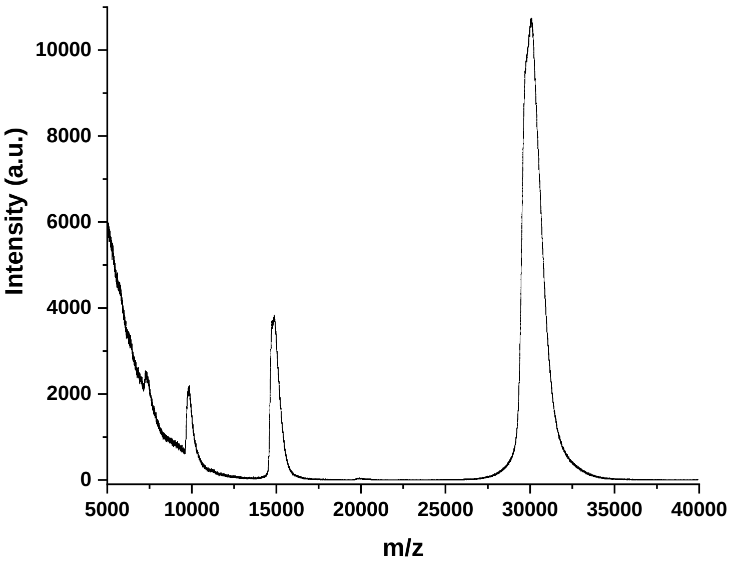


**Fig. S31.** MALDI analysis (PNA matrix, $D_2O$, pD 7.2 phosphate buffer) of **CA** (0.1 mM) in the presence of 0.5 eq. **A8**.

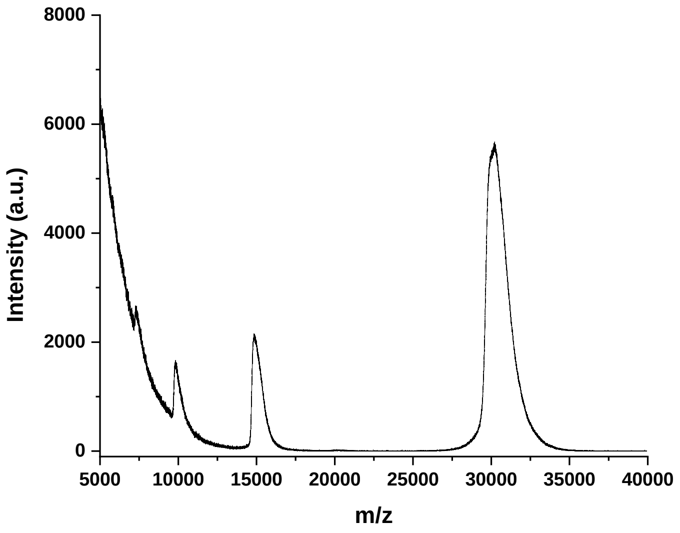


**Fig. S32.** MALDI analysis (PNA matrix, $D_2O$, pD 7.2 phosphate buffer) of **CA** (0.1 mM) in the presence of 1 eq. **A8**.

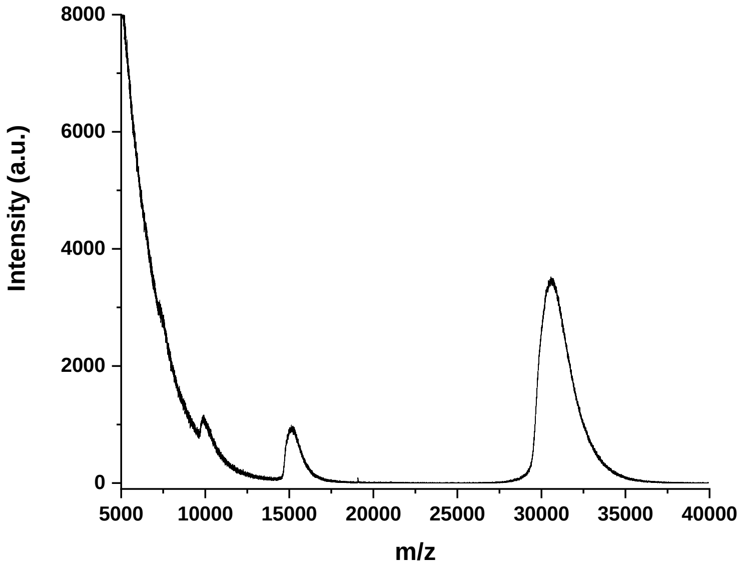


**Fig. S33.** MALDI analysis (PNA matrix, $D_2O$, pD 7.2 phosphate buffer) of **CA** (0.1 mM) in the presence of 5 eq. **A8**.

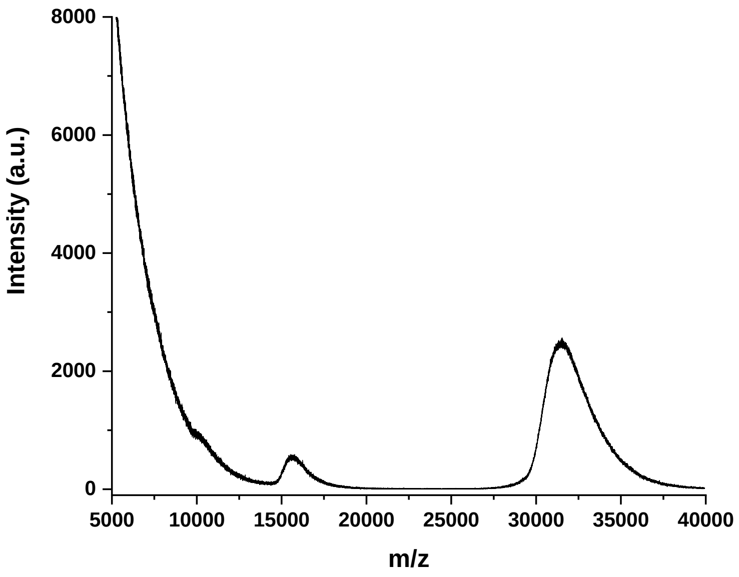


**Fig. S34.** MALDI analysis (PNA matrix, $D_2O$, pD 7.2 phosphate buffer) of **CA** (0.1 mM) in the presence of 10 eq. **A8**.

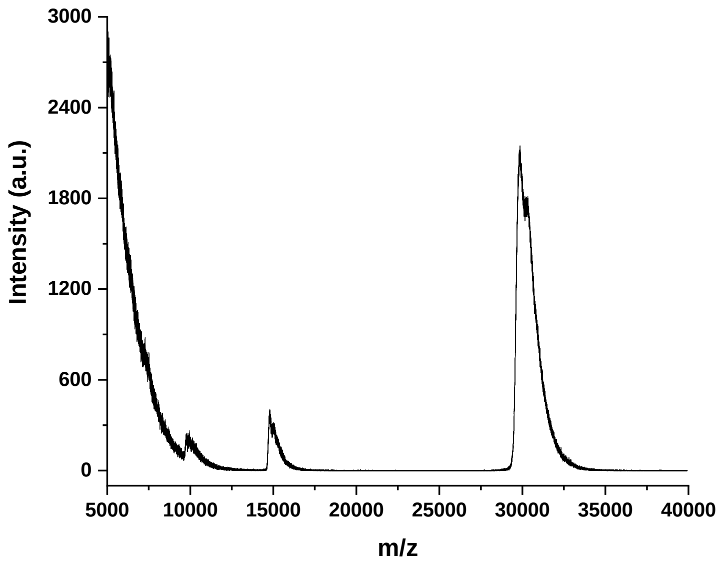


**Fig. S35.** MALDI analysis (PNA matrix, $D_2O$, pD 7.2 phosphate buffer) of **CA** (0.1 mM) in the presence of 10 eq. **A8** and 50 eq. **H1**.

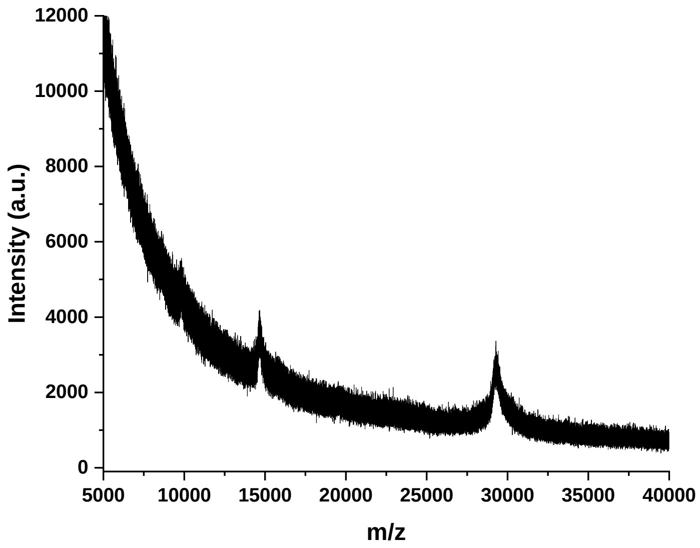


**Fig. S36.** MALDI analysis (PNA matrix, $D_2O$, pD 7.2 phosphate buffer) of **CA** (0.1 mM) in the absence of **A7**.

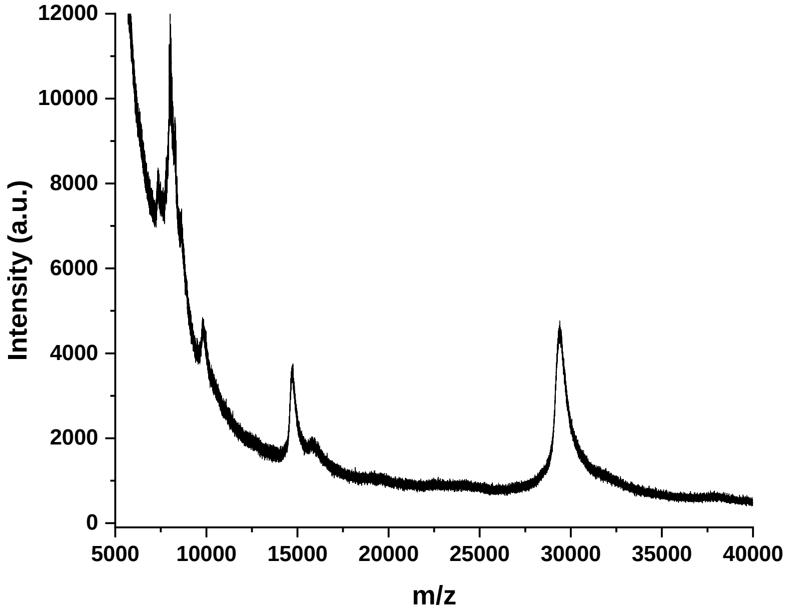


**Fig. S37.** MALDI analysis (PNA matrix, $D_2O$, pD 7.2 phosphate buffer) of **CA** (0.1 mM) in the presence of 0.5 eq. **A7**.

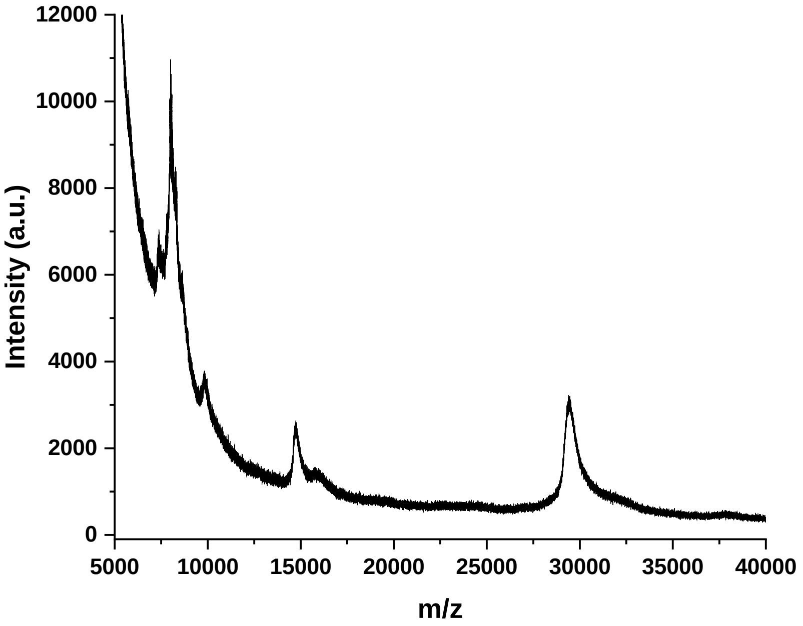


**Fig. S38.** MALDI analysis (PNA matrix, $D_2O$, pD 7.2 phosphate buffer) of **CA** (0.1 mM) in the presence of 1 eq. **A7**.

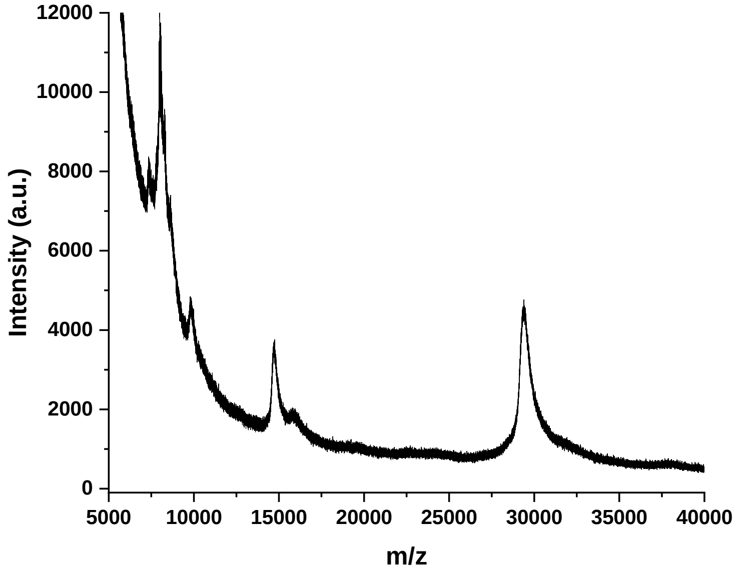


**Fig. S39.** MALDI analysis (PNA matrix, $D_2O$, pD 7.2 phosphate buffer) of **CA** (0.1 mM) in the presence of 5 eq. **A7**.

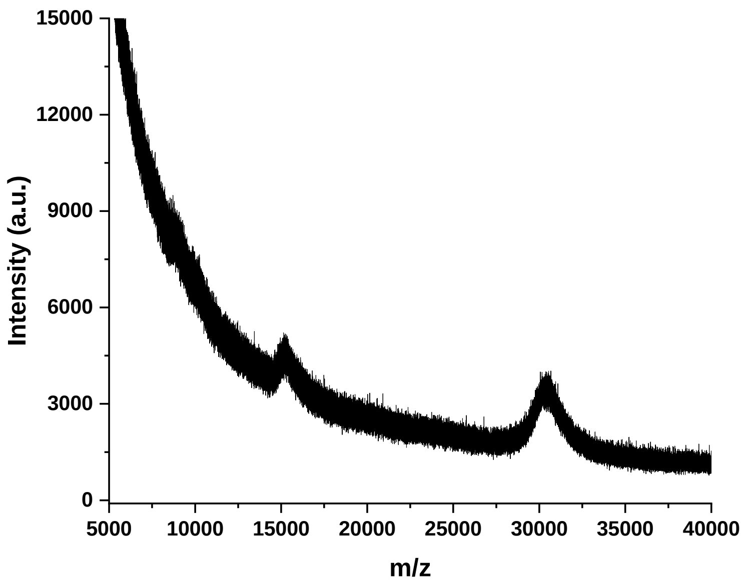


**Fig. S40.** MALDI analysis (PNA matrix, $D_2O$, pD 7.2 phosphate buffer) of **CA** (0.1 mM) in the presence of 10 eq. **A7**.

## 5. DFT cartesian coordinates and energies

- Lowest energy conformation for **$H_3O^+$**.

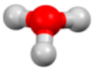

**Cartesian coordinates (4 atoms), E (298.15 K) = -76.837952 Hartree**

| | | | | |
|---|---|---|---|---|
| 1 | H | -5.2207 | -0.5771 | 0 |
| 2 | H | -5.2207 | 0.8154 | 0.8044 |
| 3 | H | -5.2207 | 0.8154 | -0.8044 |
| 4 | O | -5.5279 | 0.3513 | 0 |

- Lowest energy conformation for **A7$^{2-}$**.

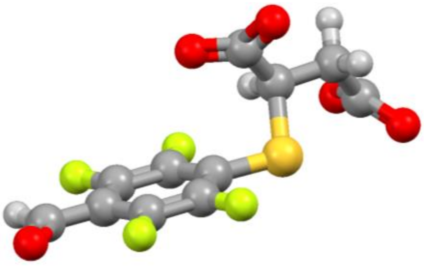

**Cartesian coordinates (25 atoms), E (298.15 K) = -1595.801763 Hartree**

| | | | | |
|---|---|---|---|---|
| 1 | C | 0.9259 | 2.5797 | -1.5674 |
| 2 | C | 1.18 | 1.4039 | -0.8812 |
| 3 | C | 0.2585 | 0.8544 | 0.0162 |
| 4 | C | -0.9321 | 1.5619 | 0.2081 |
| 5 | C | -1.1936 | 2.7254 | -0.4874 |
| 6 | C | -0.2763 | 3.2766 | -1.3935 |
| 7 | C | -0.6062 | 4.5312 | -2.1011 |
| 8 | O | 0.1151 | 5.0963 | -2.8968 |
| 9 | S | 0.6537 | -0.6348 | 0.8851 |
| 10 | C | -0.8672 | -1.7065 | 0.5896 |
| 11 | C | -1.1187 | -1.7861 | -0.95 |
| 12 | C | -0.5987 | -3.0327 | 1.2904 |
| 13 | C | -0.4499 | -2.8772 | 2.8278 |
| 14 | O | 0.667 | -3.1772 | 3.322 |
| 15 | O | -1.4624 | -2.4438 | 3.4349 |
| 16 | O | -0.6251 | -2.7617 | -1.5595 |
| 17 | O | -1.7928 | -0.8368 | -1.4207 |
| 18 | F | 1.8614 | 3.0232 | -2.4161 |
| 19 | F | -2.3652 | 3.3499 | -0.2656 |
| 20 | F | -1.8457 | 1.1296 | 1.0887 |
| 21 | F | 2.3487 | 0.7801 | -1.1109 |
| 22 | H | -1.599 | 4.9383 | -1.8458 |
| 23 | H | -1.6951 | -1.1978 | 1.0757 |
| 24 | H | -1.4706 | -3.6641 | 1.0857 |
| 25 | H | 0.2739 | -3.5214 | 0.8548 |

- Lowest energy conformation for **A8**$^{-}$.

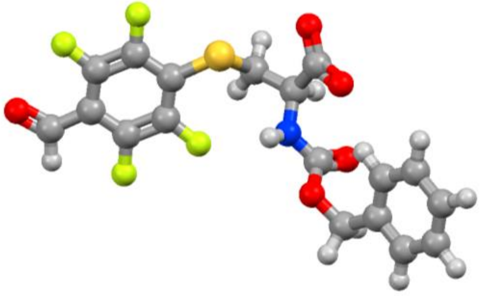

**Cartesian coordinates (41 atoms), E (298.15 K) = -1921.974968 Hartree**

| | | | | |
|---|---|---|---|---|
| 1 | C | -1.4373 | 3.5527 | -4.3547 |
| 2 | C | -0.7879 | 2.3306 | -4.4147 |
| 3 | C | -1.0473 | 1.3063 | -3.4956 |
| 4 | C | -1.9827 | 1.5852 | -2.4945 |
| 5 | C | -2.6448 | 2.7984 | -2.4434 |
| 6 | C | -2.3913 | 3.8218 | -3.3652 |
| 7 | F | -1.1325 | 4.4662 | -5.2817 |
| 8 | F | -3.5358 | 2.9937 | -1.4576 |
| 9 | F | 0.1083 | 2.1403 | -5.3961 |
| 10 | F | -2.2539 | 0.6705 | -1.5497 |
| 11 | C | -3.1163 | 5.1084 | -3.2538 |
| 12 | S | -0.112 | -0.1996 | -3.5999 |
| 13 | O | -2.9661 | 6.0572 | -3.993 |
| 14 | C | -1.4064 | -1.5354 | -3.4255 |
| 15 | C | -1.0315 | -2.5535 | -2.3554 |
| 16 | N | -1.2457 | -2.0423 | -1.0108 |
| 17 | C | 0.425 | -3.1295 | -2.5458 |
| 18 | C | -1.6226 | -2.8769 | -0.014 |
| 19 | O | 0.9781 | -3.5875 | -1.5233 |
| 20 | O | 0.865 | -3.0825 | -3.7195 |
| 21 | O | -1.3169 | -2.3216 | 1.1974 |
| 22 | O | -2.1827 | -3.9503 | -0.1454 |
| 23 | C | -1.5509 | -3.135 | 2.3567 |
| 24 | C | -0.3513 | -3.9865 | 2.7234 |
| 25 | C | 0.6781 | -4.2471 | 1.8157 |
| 26 | C | 1.75 | -5.0606 | 2.1898 |
| 27 | C | 1.8062 | -5.619 | 3.4659 |
| 28 | C | 0.7789 | -5.3598 | 4.3745 |
| 29 | C | -0.2911 | -4.5475 | 4.0043 |
| 30 | H | -3.8316 | 5.1461 | -2.4153 |
| 31 | H | -2.3654 | -1.0681 | -3.2168 |
| 32 | H | -1.4329 | -2.0228 | -4.396 |
| 33 | H | -1.713 | -3.4008 | -2.4725 |
| 34 | H | -0.6576 | -1.2708 | -0.7287 |
| 35 | H | -1.7837 | -2.4342 | 3.1607 |
| 36 | H | -2.4238 | -3.7697 | 2.1822 |
| 37 | H | 0.6569 | -3.8281 | 0.8156 |

| | | | | |
|---|---|---|---|---|
| 38 | H | 2.5426 | -5.2565 | 1.4756 |
| 39 | H | 2.6424 | -6.248 | 3.7527 |
| 40 | H | 0.8137 | -5.785 | 5.372 |
| 41 | H | -1.0846 | -4.345 | 4.7174 |

- Lowest energy conformation for $\mathbf{T}^{4+}$.

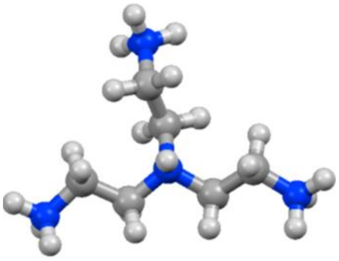

**Cartesian coordinates (32 atoms), E (298.15 K) = -460.109097 Hartree**

| | | | | |
|---|---|---|---|---|
| 1 | C | -0.6895 | -1.1699 | 0.6158 |
| 2 | C | -2.1237 | -1.1889 | 1.1462 |
| 3 | N | -2.4537 | -2.5705 | 1.6356 |
| 4 | N | -0.2477 | 0.2227 | 0.1946 |
| 5 | C | 1.2321 | 0.1782 | -0.1648 |
| 6 | C | 1.8427 | 1.5809 | -0.1702 |
| 7 | N | 3.3052 | 1.4981 | -0.4999 |
| 8 | C | -1.0921 | 0.8062 | -0.9389 |
| 9 | C | -2.1014 | 1.8331 | -0.4179 |
| 10 | N | -2.9251 | 2.3618 | -1.5566 |
| 11 | H | -2.411 | -3.2717 | 0.8893 |
| 12 | H | -3.4903 | 1.6325 | -2.0019 |
| 13 | H | 3.8203 | 0.899 | 0.1517 |
| 14 | H | -3.407 | -2.5909 | 2.0095 |
| 15 | H | -3.5757 | 3.077 | -1.2167 |
| 16 | H | 3.7305 | 2.429 | -0.4467 |
| 17 | H | -0.332 | 0.8212 | 1.0225 |
| 18 | H | -1.8313 | -2.8785 | 2.3891 |
| 19 | H | -2.3539 | 2.8032 | -2.2842 |
| 20 | H | 3.4749 | 1.1477 | -1.4477 |
| 21 | H | -0.5664 | -1.8151 | -0.2536 |
| 22 | H | 0.0025 | -1.4855 | 1.395 |
| 23 | H | -2.2489 | -0.5201 | 1.9966 |
| 24 | H | -2.861 | -0.9537 | 0.3824 |
| 25 | H | 1.717 | -0.4403 | 0.5888 |
| 26 | H | 1.3061 | -0.3178 | -1.1315 |
| 27 | H | 1.3958 | 2.2391 | -0.9117 |
| 28 | H | 1.7655 | 2.0495 | 0.8099 |
| 29 | H | -1.574 | -0.0287 | -1.4445 |
| 30 | H | -0.4141 | 1.2689 | -1.6511 |
| 31 | H | -1.6061 | 2.6901 | 0.0357 |
| 32 | H | -2.8012 | 1.4114 | 0.2999 |

- Lowest energy conformation for **B2$^{2+}$**.

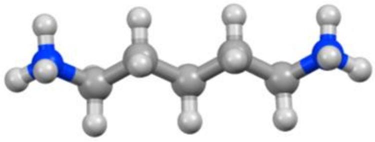

**Cartesian coordinates (23 atoms), E (298.15 K) = -309.298098 Hartree**

| | | | | |
|---|---|---|---|---|
| 1 | C | -2.5316 | -0.225 | -0.0235 |
| 2 | C | -1.2124 | 0.534 | -0.0008 |
| 3 | C | -0.0254 | -0.4363 | 0.0026 |
| 4 | C | 1.3178 | 0.3027 | -0.009 |
| 5 | C | 2.4767 | -0.6837 | 0.0212 |
| 6 | N | 3.8057 | 0.0353 | -0.0067 |
| 7 | N | -3.7062 | 0.7251 | 0.0154 |
| 8 | H | -2.6449 | -0.816 | -0.9316 |
| 9 | H | -2.6395 | -0.8809 | 0.8396 |
| 10 | H | -1.1689 | 1.1702 | 0.8892 |
| 11 | H | -1.1515 | 1.1894 | -0.8759 |
| 12 | H | -0.0865 | -1.0938 | -0.871 |
| 13 | H | -0.0823 | -1.0779 | 0.8883 |
| 14 | H | 1.38 | 0.9699 | 0.857 |
| 15 | H | 1.3875 | 0.9254 | -0.907 |
| 16 | H | 2.4689 | -1.3463 | -0.8435 |
| 17 | H | 2.473 | -1.2878 | 0.9278 |
| 18 | H | 4.5858 | -0.6255 | 0.0146 |
| 19 | H | 3.9119 | 0.6602 | 0.7963 |
| 20 | H | 3.9086 | 0.602 | -0.8522 |
| 21 | H | -4.5952 | 0.2215 | -0.0272 |
| 22 | H | -3.7105 | 1.281 | 0.8741 |
| 23 | H | -3.6881 | 1.3796 | -0.7707 |

- Lowest energy conformation for **B3$^{+}$**.

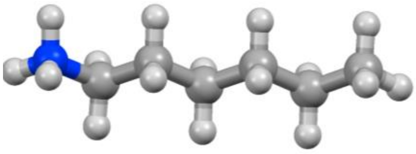

**Cartesian coordinates (23 atoms), E (298.15 K) = -292.806275 Hartree**

| | | | | |
|---|---|---|---|---|
| 1 | C | -2.5936 | -0.2443 | -0.0207 |
| 2 | C | -1.2719 | 0.5089 | 0.0047 |
| 3 | C | -0.0823 | -0.4588 | 0.0061 |
| 4 | C | 1.267 | 0.2661 | -0.0044 |
| 5 | C | 2.4629 | -0.6911 | 0.0177 |
| 6 | C | 3.8095 | 0.0371 | -0.0101 |
| 7 | N | -3.7668 | 0.7099 | 0.0094 |
| 8 | H | -2.7058 | -0.8382 | -0.9272 |
| 9 | H | -2.7081 | -0.8969 | 0.8442 |
| 10 | H | -1.2286 | 1.1445 | 0.8959 |
| 11 | H | -1.2101 | 1.1676 | -0.8684 |
| 12 | H | -0.1471 | -1.1173 | -0.8681 |
| 13 | H | -0.1421 | -1.1054 | 0.8895 |

| | | | | |
|---|---|---|---|---|
| 14 | H | 1.3251 | 0.9387 | 0.8605 |
| 15 | H | 1.3286 | 0.9032 | -0.8955 |
| 16 | H | 2.3955 | -1.3713 | -0.8398 |
| 17 | H | 2.4034 | -1.3202 | 0.914 |
| 18 | H | 4.6453 | -0.6677 | 0.0111 |
| 19 | H | 3.9127 | 0.7045 | 0.8514 |
| 20 | H | 3.9077 | 0.6455 | -0.9147 |
| 21 | H | -4.6581 | 0.2108 | -0.0334 |
| 22 | H | -3.7704 | 1.2707 | 0.8649 |
| 23 | H | -3.7417 | 1.3602 | -0.7799 |

- Lowest energy conformation for **B4**$^-$.

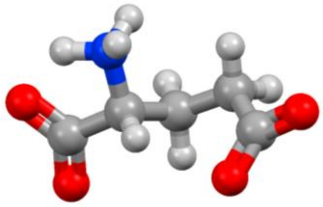

**Cartesian coordinates (18 atoms), E (298.15 K) = -551.240975 Hartree**

| | | | | |
|---|---|---|---|---|
| 1 | N | -1.8498 | 0.1635 | -0.8359 |
| 2 | C | -0.517 | 0.7872 | -0.4769 |
| 3 | C | -0.8331 | 2.2976 | -0.2318 |
| 4 | C | 0.15 | 0.0922 | 0.7035 |
| 5 | O | 0.1059 | 3.0036 | 0.1621 |
| 6 | O | -2.0297 | 2.632 | -0.4806 |
| 7 | C | 0.5372 | -1.3635 | 0.4347 |
| 8 | C | 1.5994 | -1.5882 | -0.6823 |
| 9 | O | 1.8329 | -2.7883 | -0.9621 |
| 10 | O | 2.1315 | -0.5676 | -1.1926 |
| 11 | H | -1.8477 | -0.336 | -1.7223 |
| 12 | H | -2.2063 | -0.4604 | -0.1133 |
| 13 | H | -2.4495 | 1.0375 | -0.8816 |
| 14 | H | 0.1369 | 0.6932 | -1.3429 |
| 15 | H | 1.0449 | 0.6758 | 0.9181 |
| 16 | H | -0.5035 | 0.1659 | 1.5826 |
| 17 | H | 0.9384 | -1.8059 | 1.3531 |
| 18 | H | -0.3388 | -1.9741 | 0.1845 |

- Lowest energy conformation for **KK**$^+$.

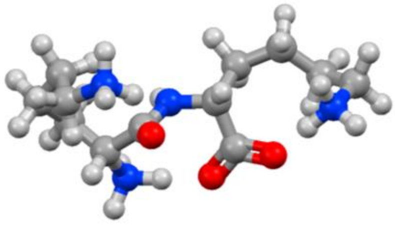

**Cartesian coordinates (47 atoms), E (298.15 K) = -918.511927 Hartree**

| | | | | |
|---|---|---|---|---|
| 1 | N | -0.9536 | -1.2815 | 2.5909 |
| 2 | C | -1.6048 | -0.0988 | 1.8846 |
| 3 | C | -0.6304 | 0.2977 | 0.7642 |

| | | | | |
|---|---|---|---|---|
| 4 | C | -3.0313 | -0.4459 | 1.4379 |
| 5 | O | -0.1174 | 1.4148 | 0.7452 |
| 6 | C | -3.906 | 0.7538 | 1.0294 |
| 7 | C | -3.3404 | 1.6496 | -0.0973 |
| 8 | C | -2.6891 | 2.9271 | 0.4482 |
| 9 | N | -1.6088 | 3.4294 | -0.4756 |
| 10 | N | -0.3204 | -0.6735 | -0.1207 |
| 11 | C | 1.0465 | -0.7527 | -0.65 |
| 12 | C | 2.0249 | -0.9968 | 0.5474 |
| 13 | C | 1.0778 | -1.8364 | -1.7505 |
| 14 | O | 3.2487 | -0.8158 | 0.3321 |
| 15 | O | 1.5439 | -1.3556 | 1.6529 |
| 16 | C | 2.2395 | -1.7626 | -2.756 |
| 17 | C | 3.5754 | -2.3947 | -2.2934 |
| 18 | C | 4.8026 | -1.5183 | -2.5248 |
| 19 | N | 4.6647 | -0.2012 | -1.794 |
| 20 | H | -0.9825 | -1.1613 | 3.6035 |
| 21 | H | -1.432 | -2.1596 | 2.3833 |
| 22 | H | 0.0716 | -1.3778 | 2.2725 |
| 23 | H | -1.6078 | 0.7207 | 2.5997 |
| 24 | H | -2.9875 | -1.165 | 0.6134 |
| 25 | H | -3.5257 | -0.953 | 2.2712 |
| 26 | H | -4.8653 | 0.3432 | 0.7101 |
| 27 | H | -4.1198 | 1.3616 | 1.9143 |
| 28 | H | -2.623 | 1.0836 | -0.6996 |
| 29 | H | -4.1407 | 1.9333 | -0.7829 |
| 30 | H | -3.4179 | 3.7265 | 0.5682 |
| 31 | H | -2.1912 | 2.7649 | 1.4013 |
| 32 | H | -1.281 | 4.3574 | -0.2015 |
| 33 | H | -0.8062 | 2.7834 | -0.4132 |
| 34 | H | -1.9232 | 3.4861 | -1.446 |
| 35 | H | -0.786 | -1.5657 | -0.0203 |
| 36 | H | 1.2942 | 0.2167 | -1.0838 |
| 37 | H | 1.0397 | -2.829 | -1.288 |
| 38 | H | 0.1465 | -1.718 | -2.3111 |
| 39 | H | 1.9167 | -2.2647 | -3.6705 |
| 40 | H | 2.3742 | -0.7148 | -3.0454 |
| 41 | H | 3.5318 | -2.6481 | -1.234 |
| 42 | H | 3.7496 | -3.3307 | -2.8289 |
| 43 | H | 5.6974 | -2.0019 | -2.136 |
| 44 | H | 4.9602 | -1.2963 | -3.58 |
| 45 | H | 5.574 | 0.1797 | -1.5357 |
| 46 | H | 4.2021 | 0.5026 | -2.3676 |
| 47 | H | 4.0736 | -0.3489 | -0.9101 |

- Lowest energy conformation for **A7T⁺**.

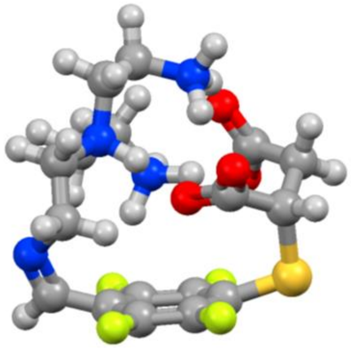

**Cartesian coordinates (53 atoms), E (298.15 K) = -1979.133369 Hartree**

| | | | | |
|---|---|---|---|---|
| 1 | C | 1.6535 | 3.2178 | -1.4843 |
| 2 | C | -0.5315 | 3.7726 | -0.6406 |
| 3 | C | 0.0851 | 4.1564 | 0.5504 |
| 4 | C | 1.4485 | 3.9986 | 0.7401 |
| 5 | C | 2.2462 | 3.4346 | -0.2465 |
| 6 | C | 3.5824 | 2.8186 | 0.0173 |
| 7 | F | 2.3966 | 2.6603 | -2.4779 |
| 8 | F | -0.6532 | 4.5395 | 1.6012 |
| 9 | F | 1.9717 | 4.2619 | 1.9516 |
| 10 | S | -2.2941 | 3.5662 | -0.7211 |
| 11 | C | -2.5808 | 1.9772 | 0.2608 |
| 12 | C | -3.0317 | 0.7825 | -0.626 |
| 13 | C | -1.4428 | 1.457 | 1.1671 |
| 14 | O | -0.3069 | 1.2889 | 0.6466 |
| 15 | O | -1.7887 | 1.0861 | 2.3125 |
| 16 | C | -1.863 | 0.0611 | -1.2963 |
| 17 | O | -1.4013 | 0.6009 | -2.3409 |
| 18 | O | -1.4009 | -0.9944 | -0.7816 |
| 19 | C | 0.2973 | 3.3886 | -1.6941 |
| 20 | F | -0.1987 | 3.0718 | -2.8936 |
| 21 | N | 3.789 | 1.8572 | 0.8228 |
| 22 | C | 2.7348 | 1.3612 | 1.7069 |
| 23 | C | 2.5323 | -0.1507 | 1.6423 |
| 24 | N | 1.5968 | -0.6305 | 0.5366 |
| 25 | C | 2.3397 | -0.6404 | -0.7868 |
| 26 | C | 1.5908 | -1.1131 | -2.0383 |
| 27 | C | 1.0806 | -2.0166 | 0.8576 |
| 28 | C | 0.1523 | -2.1425 | 2.0585 |
| 29 | N | -1.1853 | -1.5052 | 1.8476 |
| 30 | H | 4.4118 | 3.1448 | -0.6078 |
| 31 | H | -3.4153 | 2.2564 | 0.9002 |
| 32 | H | -3.56 | 0.0827 | 0.0261 |
| 33 | H | -3.7266 | 1.1347 | -1.3895 |
| 34 | H | 3.0799 | 1.5688 | 2.7237 |
| 35 | H | 1.7609 | 1.8386 | 1.5921 |
| 36 | H | 3.4743 | -0.6866 | 1.5267 |
| 37 | H | 2.0741 | -0.4556 | 2.5782 |

| | | | | |
|---|---|---|---|---|
| 38 | H | 0.7867 | 0.05 | 0.5055 |
| 39 | H | 2.7425 | 0.3594 | -0.9292 |
| 40 | H | 3.1877 | -1.3062 | -0.6316 |
| 41 | H | 2.3164 | -1.6444 | -2.6515 |
| 42 | H | 0.7725 | -1.7924 | -1.815 |
| 43 | H | 0.5584 | -2.3801 | -0.021 |
| 44 | H | 1.9547 | -2.6456 | 1.0288 |
| 45 | H | -0.0034 | -3.2109 | 2.2009 |
| 46 | H | 0.5758 | -1.7507 | 2.9805 |
| 47 | H | -1.2507 | -0.5294 | 2.2358 |
| 48 | H | -1.4221 | -1.4063 | 0.8173 |
| 49 | H | -1.9178 | -2.0499 | 2.3004 |
| 50 | H | -0.0662 | 0.2459 | -2.5889 |
| 51 | H | 1.588 | 0.8158 | -2.8862 |
| 52 | H | 0.9446 | -0.3375 | -3.8398 |
| 53 | N | 1.0039 | -0.0202 | -2.8722 |

- Lowest energy conformation for **A7B2**$^-$.

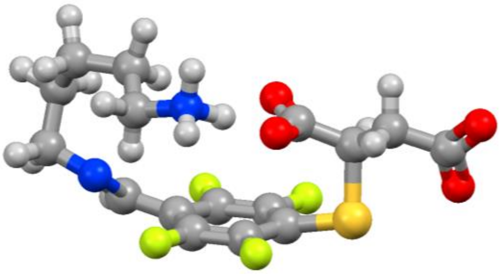

**Cartesian coordinates (44 atoms), E (298.15 K) = -1828.231057 Hartree**

| | | | | |
|---|---|---|---|---|
| 1 | C | -0.5898 | 1.4461 | 2.3716 |
| 2 | C | 0.1579 | 0.3091 | 2.6268 |
| 3 | C | -0.3794 | -0.9679 | 2.4618 |
| 4 | C | -1.7341 | -1.0479 | 2.1456 |
| 5 | C | -2.4866 | 0.0901 | 1.9125 |
| 6 | C | -1.9218 | 1.364 | 1.9587 |
| 7 | C | -2.675 | 2.4928 | 1.3795 |
| 8 | N | -2.0662 | 3.3938 | 0.7288 |
| 9 | S | 0.6509 | -2.4297 | 2.4425 |
| 10 | C | 0.7825 | -2.6146 | 0.5592 |
| 11 | C | 0.518 | -1.2579 | -0.1463 |
| 12 | C | 2.1509 | -3.1805 | 0.1926 |
| 13 | C | 2.5435 | -4.5451 | 0.8156 |
| 14 | O | 1.8997 | -4.9187 | 1.8322 |
| 15 | O | 3.4936 | -5.1414 | 0.2576 |
| 16 | O | 1.4419 | -0.3878 | -0.0218 |
| 17 | O | -0.5457 | -1.124 | -0.7697 |
| 18 | F | 0.0242 | 2.6393 | 2.4762 |
| 19 | F | -3.7706 | -0.0523 | 1.5227 |
| 20 | F | -2.3213 | -2.2417 | 1.9619 |
| 21 | F | 1.4452 | 0.47 | 2.9666 |
| 22 | C | -2.8467 | 4.3268 | -0.0626 |

| | | | | |
|---|---|---|---|---|
| 23 | C | -2.8699 | 3.868 | -1.5335 |
| 24 | C | -1.5262 | 3.8159 | -2.2825 |
| 25 | C | -0.5555 | 2.6501 | -1.9639 |
| 26 | C | 0.5415 | 3.0181 | -0.9668 |
| 27 | N | 1.6389 | 1.9944 | -0.9506 |
| 28 | H | -3.7677 | 2.4305 | 1.4374 |
| 29 | H | -0.013 | -3.3034 | 0.2795 |
| 30 | H | 2.1904 | -3.2877 | -0.8973 |
| 31 | H | 2.9177 | -2.445 | 0.4537 |
| 32 | H | -3.8826 | 4.4128 | 0.2978 |
| 33 | H | -2.3771 | 5.313 | 0.0034 |
| 34 | H | -3.3513 | 2.8844 | -1.5791 |
| 35 | H | -3.5273 | 4.5621 | -2.0677 |
| 36 | H | -1.7693 | 3.7534 | -3.347 |
| 37 | H | -1.0057 | 4.7729 | -2.1565 |
| 38 | H | -1.1001 | 1.773 | -1.6029 |
| 39 | H | -0.0718 | 2.3509 | -2.9007 |
| 40 | H | 0.9976 | 3.9721 | -1.2391 |
| 41 | H | 0.1457 | 3.0965 | 0.0426 |
| 42 | H | 2.0482 | 1.8891 | -1.8794 |
| 43 | H | 2.3924 | 2.3021 | -0.3358 |
| 44 | H | 1.37 | 0.9752 | -0.5937 |

- Lowest energy conformation for **A7B3**$^{-2}$.

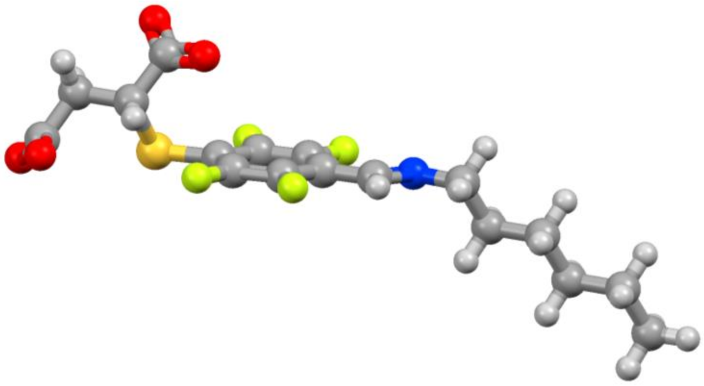

**Cartesian coordinates (44 atoms), E (298.15 K) = -1811.711591 Hartree**

| | | | | |
|---|---|---|---|---|
| 1 | C | 0.5491 | -1.7566 | -0.7936 |
| 2 | C | 0.9988 | -2.9267 | -0.1984 |
| 3 | C | 0.3022 | -3.5506 | 0.8377 |
| 4 | C | -0.8775 | -2.931 | 1.2524 |
| 5 | C | -1.3352 | -1.775 | 0.6498 |
| 6 | C | -0.6404 | -1.1391 | -0.3886 |
| 7 | C | -1.1874 | 0.1018 | -0.9588 |
| 8 | N | -0.6328 | 0.7568 | -1.896 |
| 9 | S | 0.9305 | -5.0248 | 1.5961 |
| 10 | C | -0.515 | -6.2156 | 1.3978 |
| 11 | C | -0.9098 | -6.2666 | -0.111 |
| 12 | C | -0.0768 | -7.539 | 2.0139 |

| | | | | |
|---|---|---|---|---|
| 13 | C | 0.1697 | -7.4368 | 3.5433 |
| 14 | O | 1.335 | -7.6775 | 3.9513 |
| 15 | O | -0.8269 | -7.1047 | 4.2351 |
| 16 | O | -0.3659 | -7.1487 | -0.8149 |
| 17 | O | -1.7386 | -5.393 | -0.4676 |
| 18 | F | 1.2943 | -1.2441 | -1.7839 |
| 19 | F | -2.4895 | -1.2415 | 1.1015 |
| 20 | F | -1.5981 | -3.4484 | 2.2605 |
| 21 | F | 2.1435 | -3.4625 | -0.661 |
| 22 | C | -1.2864 | 1.9808 | -2.3365 |
| 23 | C | -0.3623 | 3.1876 | -2.1331 |
| 24 | C | -0.9618 | 4.4888 | -2.6735 |
| 25 | C | -0.0437 | 5.6988 | -2.4749 |
| 26 | C | -0.6326 | 7.0057 | -3.0157 |
| 27 | C | 0.2954 | 8.2074 | -2.8158 |
| 28 | H | -2.13 | 0.4296 | -0.5056 |
| 29 | H | -1.3301 | -5.795 | 1.9816 |
| 30 | H | -0.9014 | -8.2403 | 1.8425 |
| 31 | H | 0.7999 | -7.9278 | 1.4931 |
| 32 | H | -1.4983 | 1.8737 | -3.407 |
| 33 | H | -2.2457 | 2.1512 | -1.8222 |
| 34 | H | -0.1508 | 3.2932 | -1.0626 |
| 35 | H | 0.5954 | 2.9848 | -2.6249 |
| 36 | H | -1.1798 | 4.3709 | -3.7425 |
| 37 | H | -1.9241 | 4.6801 | -2.1822 |
| 38 | H | 0.1751 | 5.8162 | -1.4057 |
| 39 | H | 0.9193 | 5.5064 | -2.9653 |
| 40 | H | -0.8535 | 6.8859 | -4.0833 |
| 41 | H | -1.5937 | 7.1982 | -2.5237 |
| 42 | H | -0.1486 | 9.1269 | -3.2076 |
| 43 | H | 0.5091 | 8.3652 | -1.7538 |
| 44 | H | 1.2514 | 8.0537 | -3.3265 |

- Lowest energy conformation for **A7B4$^{-4}$**.

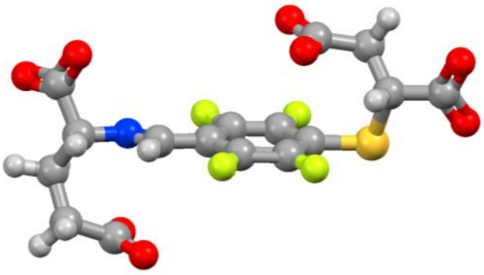

**Cartesian coordinates (39 atoms), E (298.15 K) = -2070.120657999 Hartree**

| | | | | |
|---|---|---|---|---|
| 1 | C | 0.6457 | -0.1295 | -0.6324 |
| 2 | C | 0.7973 | -1.5079 | -0.6486 |
| 3 | C | -0.2219 | -2.373 | -0.2544 |
| 4 | C | -1.4126 | -1.7777 | 0.1586 |
| 5 | C | -1.5609 | -0.4047 | 0.1904 |

| | | | | |
|---|---|---|---|---|
| 6 | C | -0.5434 | 0.4731 | -0.2057 |
| 7 | C | -0.7939 | 1.9217 | -0.1593 |
| 8 | S | -0.0133 | -4.1466 | -0.2759 |
| 9 | C | 0.0083 | -4.5152 | 1.5573 |
| 10 | C | 0.0163 | -6.0666 | 1.7072 |
| 11 | C | 1.1639 | -3.8352 | 2.2887 |
| 12 | C | 0.9499 | -2.3261 | 2.5937 |
| 13 | O | -0.1923 | -2.0046 | 3.0121 |
| 14 | O | 1.9389 | -1.5703 | 2.4248 |
| 15 | O | -0.0329 | -6.4702 | 2.8914 |
| 16 | O | 0.0683 | -6.7553 | 0.6581 |
| 17 | F | 1.7005 | 0.6092 | -1.0177 |
| 18 | F | -2.7412 | 0.0955 | 0.6215 |
| 19 | F | -2.4459 | -2.539 | 0.5614 |
| 20 | F | 1.9865 | -1.9995 | -1.0437 |
| 21 | N | 0.0211 | 2.7958 | -0.5881 |
| 22 | C | -0.3666 | 4.1896 | -0.4834 |
| 23 | C | 0.598 | 4.8696 | 0.5713 |
| 24 | C | -0.3546 | 4.8489 | -1.8661 |
| 25 | O | 0.498 | 4.4096 | 1.7329 |
| 26 | O | 1.3452 | 5.7894 | 0.166 |
| 27 | C | -1.479 | 4.3603 | -2.7914 |
| 28 | C | -1.2647 | 2.9368 | -3.376 |
| 29 | O | -2.1868 | 2.0979 | -3.2028 |
| 30 | O | -0.187 | 2.7553 | -4.0008 |
| 31 | H | -1.7684 | 2.1999 | 0.2542 |
| 32 | H | -0.9229 | -4.1269 | 1.9728 |
| 33 | H | 1.2396 | -4.3463 | 3.2553 |
| 34 | H | 2.1066 | -3.9839 | 1.7561 |
| 35 | H | -1.3823 | 4.2789 | -0.0572 |
| 36 | H | -0.4286 | 5.9285 | -1.7075 |
| 37 | H | 0.6092 | 4.6625 | -2.3459 |
| 38 | H | -2.4412 | 4.3977 | -2.2695 |
| 39 | H | -1.5458 | 5.0457 | -3.6464 |

- Lowest energy conformation for **A7KK**$^-$.

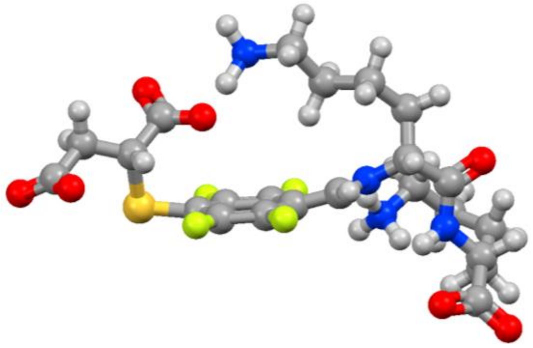

**Cartesian coordinates (68 atoms), E (298.15 K) = -2437.447269 Hartree**

| | | | | |
|---|---|---|---|---|
| 1 | C | 1.1432 | 0.5719 | 2.1565 |
| 2 | C | 1.8628 | -0.4822 | 2.6904 |

| | | | | |
|---|---|---|---|---|
| 3 | C | 1.2367 | -1.504 | 3.403 |
| 4 | C | -0.1343 | -1.3777 | 3.623 |
| 5 | C | -0.8532 | -0.3282 | 3.0836 |
| 6 | C | -0.2463 | 0.6653 | 2.3028 |
| 7 | C | -1.1062 | 1.572 | 1.5412 |
| 8 | S | 2.1187 | -2.9785 | 3.8466 |
| 9 | C | 1.2395 | -4.1933 | 2.703 |
| 10 | C | 1.337 | -3.6669 | 1.258 |
| 11 | C | 1.8627 | -5.5607 | 2.9387 |
| 12 | C | 1.5368 | -6.1421 | 4.3464 |
| 13 | O | 0.3297 | -6.0873 | 4.6927 |
| 14 | O | 2.5005 | -6.6197 | 4.9926 |
| 15 | O | 2.2562 | -4.0343 | 0.5181 |
| 16 | O | 0.4043 | -2.8393 | 0.9377 |
| 17 | F | 1.8233 | 1.4629 | 1.4114 |
| 18 | F | -2.184 | -0.3021 | 3.2714 |
| 19 | F | -0.8004 | -2.3251 | 4.2977 |
| 20 | F | 3.1769 | -0.5403 | 2.4339 |
| 21 | N | -0.6719 | 2.4467 | 0.7209 |
| 22 | C | -1.6352 | 3.0584 | -0.1881 |
| 23 | C | -1.5562 | 4.603 | -0.1226 |
| 24 | C | -1.3647 | 2.5569 | -1.6198 |
| 25 | O | -1.6924 | 5.2916 | -1.1289 |
| 26 | C | -1.5141 | 1.0371 | -1.8192 |
| 27 | C | -0.2619 | 0.1973 | -1.5132 |
| 28 | C | -0.453 | -1.255 | -1.9423 |
| 29 | N | 0.6823 | -2.112 | -1.4799 |
| 30 | N | -1.2982 | 5.1035 | 1.1093 |
| 31 | C | -1.4076 | 6.5224 | 1.4602 |
| 32 | C | -2.0359 | 6.6099 | 2.894 |
| 33 | C | -0.0564 | 7.2613 | 1.4419 |
| 34 | O | -2.3004 | 7.7619 | 3.2932 |
| 35 | O | -2.1861 | 5.5174 | 3.5015 |
| 36 | C | 0.6538 | 7.3693 | 0.0668 |
| 37 | C | 1.9376 | 6.5394 | -0.1391 |
| 38 | C | 1.7516 | 5.0379 | -0.3517 |
| 39 | N | 1.5027 | 4.2902 | 0.9342 |
| 40 | H | -2.1761 | 1.3733 | 1.6397 |
| 41 | H | 0.2019 | -4.2086 | 3.0281 |
| 42 | H | 1.4287 | -6.2348 | 2.1912 |
| 43 | H | 2.9397 | -5.5381 | 2.7641 |
| 44 | H | -2.6652 | 2.787 | 0.0914 |
| 45 | H | -2.0682 | 3.0838 | -2.266 |
| 46 | H | -0.3643 | 2.8775 | -1.9273 |
| 47 | H | -2.3571 | 0.6654 | -1.2247 |
| 48 | H | -1.7863 | 0.8657 | -2.8658 |
| 49 | H | 0.5984 | 0.6196 | -2.0446 |
| 50 | H | -0.0162 | 0.2234 | -0.4516 |

| | | | | |
|---|---|---|---|---|
| 51 | H | -1.3597 | -1.6763 | -1.5032 |
| 52 | H | -0.5357 | -1.3289 | -3.0281 |
| 53 | H | 0.7128 | -2.991 | -1.9947 |
| 54 | H | 1.5808 | -1.6553 | -1.6324 |
| 55 | H | 0.5882 | -2.4074 | -0.3646 |
| 56 | H | -1.5102 | 4.5313 | 1.9306 |
| 57 | H | -2.0825 | 7.0086 | 0.7513 |
| 58 | H | 0.6089 | 6.8112 | 2.1912 |
| 59 | H | -0.2713 | 8.2618 | 1.8199 |
| 60 | H | -0.0556 | 7.1238 | -0.7278 |
| 61 | H | 0.9369 | 8.4141 | -0.0862 |
| 62 | H | 2.4357 | 6.9073 | -1.0406 |
| 63 | H | 2.641 | 6.71 | 0.6839 |
| 64 | H | 2.6336 | 4.5834 | -0.7998 |
| 65 | H | 0.9 | 4.8425 | -1.0018 |
| 66 | H | 2.3642 | 4.1286 | 1.4551 |
| 67 | H | 1.0126 | 3.3834 | 0.773 |
| 68 | H | 0.8507 | 4.8096 | 1.5257 |

## 6. References


1- M.J. A Frisch, G.W.Trucks, H.B. Schlegel, G.E. Scuseria, M.A. Robb, J.R. Cheeseman, G. Scalmani, V. Barone, B. Mennucci, G.A. Petersson, et al. Gaussian 09, Revision B.01 (Gaussian, Inc), **2010**.

2- A.D. Becke, *J. Chem. Phys.,* 1993, **98**, 5648–5652.

3- S. Miertus and J. Tomasi, *Chem. Phys.,* 1982*,* **65**, 239–241.

4- A. Grosdidier, V. Zoete and O. Michielin, *Nucleic Acids Res.,* 2011, **39***,* W270-277.
